# CLUH functions as a negative regulator of inflammation in human macrophages and determines ulcerative colitis pathogenesis

**DOI:** 10.1172/jci.insight.161096

**Published:** 2023-06-08

**Authors:** Shaziya Khan, Desh Raj, Shikha Sahu, Anam Naseer, Nishakumari C. Singh, Sunaina Kumari, Sharmeen Ishteyaque, Jyotsna Sharma, Promila Lakra, Madhav N. Mugale, Arun Kumar Trivedi, Mrigank Srivastava, Tulika Chandra, Vivek Bhosale, Manoj Kumar Barthwal, Shashi Kumar Gupta, Kalyan Mitra, Aamir Nazir, Uday C. Ghoshal, Amit Lahiri

**Affiliations:** 1 Pharmacology Division, CSIR - Central Drug Research Institute, Lucknow, India.; 2 Academy of Scientific and Innovative Research (AcSIR), Ghaziabad, India.; 3 Department of Gastroenterology, Sanjay Gandhi Postgraduate Institute of Medicine, Lucknow, India.; 4 Toxicology and Experimental Medicine Division,; 5 Sophisticated Analytical Instrument Facility and Research Division,; 6 Cancer Biology Division, and; 7 Molecular Microbiology and Immunology Division, CSIR - Central Drug Research Institute, Lucknow, India.; 8 Department of Transfusion Medicine, King George’s Medical University, Lucknow, India.

**Keywords:** Gastroenterology, Autoimmune diseases

## Abstract

Altered mitochondrial function without a well-defined cause has been documented in patients with ulcerative colitis (UC). In our efforts to understand UC pathogenesis, we observed reduced expression of clustered mitochondrial homolog (CLUH) only in the active UC tissues compared with the unaffected areas from the same patient and healthy controls. Stimulation with bacterial Toll-like receptor (TLR) ligands similarly reduced CLUH expression in human primary macrophages. Further, CLUH negatively regulated secretion of proinflammatory cytokines IL-6 and TNF-α and rendered a proinflammatory niche in TLR ligand–stimulated macrophages. CLUH was further found to bind to mitochondrial fission protein dynamin related protein 1 (DRP1) and regulated DRP1 transcription in human macrophages. In the TLR ligand–stimulated macrophages, absence of CLUH led to enhanced DRP1 availability for mitochondrial fission, and a smaller dysfunctional mitochondrial pool was observed. Mechanistically, this fissioned mitochondrial pool in turn enhanced mitochondrial ROS production and reduced mitophagy and lysosomal function in CLUH-knockout macrophages. Remarkably, our studies in the mouse model of colitis with CLUH knockdown displayed exacerbated disease pathology. Taken together, this is the first report to our knowledge explaining the role of CLUH in UC pathogenesis, by means of regulating inflammation via maintaining mitochondrial-lysosomal functions in the human macrophages and intestinal mucosa.

## Graphical abstract

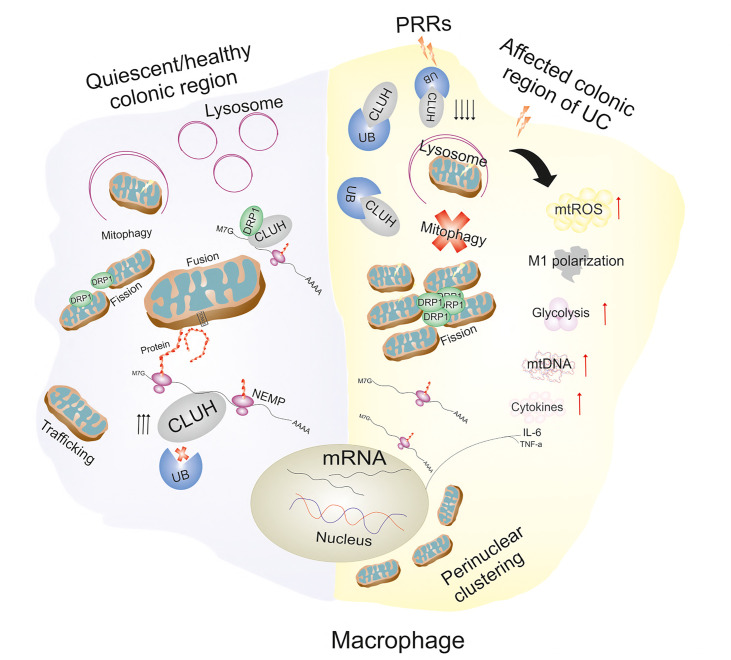

## Introduction

Ulcerative colitis (UC), an arm of inflammatory bowel disease (IBD), leads to dysregulated mucosal immune response against gut bacteria and bacterium-derived products in genetically susceptible hosts (1). Genome-wide association studies have identified more than 100 susceptibility loci in IBD (2), and alteration in cytokine production, autophagy, and antibacterial killing pathways has been highlighted as key pathogenic mechanisms, in addition to multiple environmental factors (1–4). Interestingly, alteration in mitochondrial functions like ATP synthesis, ROS production, and mitochondrial DNA (mtDNA) release is evident in patients with IBD and in the animal model of colitis (5). Mitochondria, in addition to their metabolic role, have recently emerged as a key organelle; they can regulate immune pathways and undergo continuous division, fusion, and mitophagy-mediated clearance, collectively termed as mitochondrial dynamics (6–8). In the animal model of colitis, targeting mitochondrial fission has been shown to have a therapeutic effect, indicating probable dysfunctional mitochondrial dynamics in patients with IBD (9). Further, robust colonic mitochondriopathy has recently been observed in patients with UC, which ultimately leads to dysfunction in the mitochondrial electron transport chain in the colonic mucosa (10).

Remodeling of mitochondrial biogenesis, fission, fusion, mitophagy, and trafficking pathways are highly coordinated, and alteration in these dynamics can change the metabolic and inflammatory status of immune cells including macrophages (11–14). Pathogenic bacteria and pathogen-associated molecular patterns, like lipopolysaccharide (LPS) that activates host pattern recognition receptors (PRRs), changes the mitochondrial function and dynamics in human macrophages by reducing mitochondrial oxidative phosphorylation and enhancing glycolytic pathways, allowing a “Warburg” kind of situation and a proinflammatory niche (15, 16). A subsequent break in the mitochondrial TCA cycle thereby releases mtDNA, ROS, and multiple intermediates like citrate, which ultimately leads to proinflammatory cytokine production (17, 18). LPS further has been shown to enhance mitochondrial fission and reduction in mitophagy. This change in mitochondrial dynamics downstream of PRR signaling is crucial during macrophage polarization and in multiple inflammatory and autoimmune disorders (19–22). Mitochondrial dynamics pathways and their molecular regulation in UC pathology are not well defined. Elucidating molecular mechanisms that control these dynamic changes of mitochondria in the colonic mucosa of UC patients is paramount to understand the crosstalk between environmental factors and genetic determinants that dictates IBD susceptibility.

Clustered mitochondrial homolog (CLUH) is a cellular RNA-binding protein (RBP) that specifically binds to mRNA of nuclear encoded mitochondrial proteins (NEMPs) and helps their translation and transport to mitochondria (23). In HeLa cells depleted of CLUH, mitochondrial dysfunction is documented, and mitochondria show perinuclear clustering in the absence of CLUH (24). Further, CLUH regulates mitophagy in hepatocytes and thus serves as a critical determinant of mitochondrial dynamics during starvation (25). CLUH-knockout animals cannot survive the fetal to neonatal transition (26), and human preterm babies have reduced CLUH expression (27). No study to our knowledge has yet defined the role of CLUH in PRR-induced immune function, specifically in human myeloid-derived cells and in patients with UC.

We report here for the first time to our knowledge that in quiescent cells, CLUH regulates mitochondrial dynamin related protein 1 (DRP1) protein function and allows cellular mitophagy processes to keep inflammation in check. Following LPS signaling in the affected colonic region of patients with UC, degradation of CLUH prevailed, mimicking a CLUH-knockdown condition. LPS signaling and/or CLUH knockdown in turn led to reduced mitophagy and higher glycolysis, mitochondrial ROS (mitoROS), mtDNA, and TCA cycle intermediate and proinflammatory cytokine release from the human primary macrophages. Thus, CLUH acts as a negative regulator of cytokine production in primary human macrophages and in mucosal immune cells.

## Results

### CLUH is expressed in human intestinal tissue and macrophages, and CLUH protein expression is reduced only in the disease-affected region from patients with UC.

**Figure 1:**
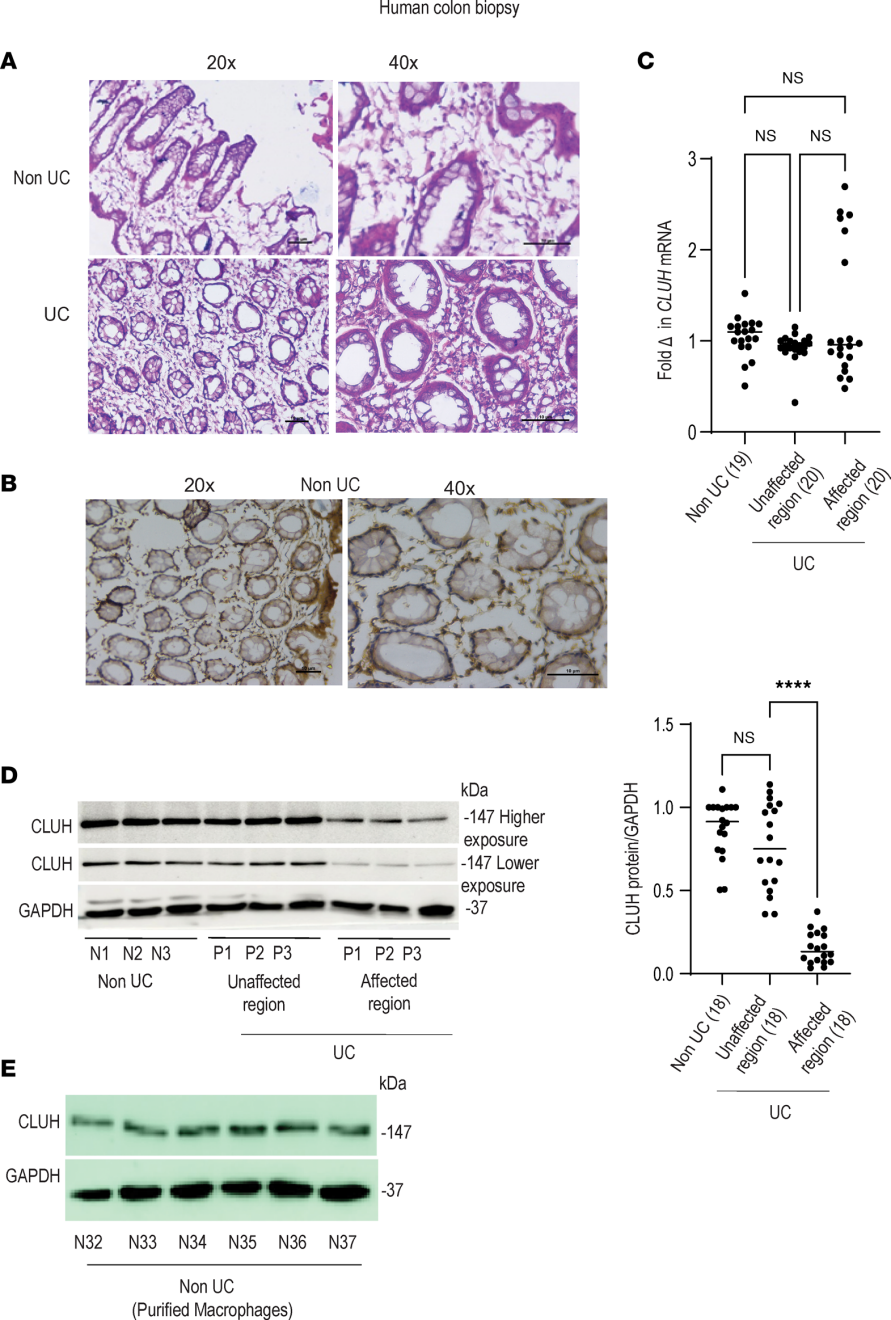
CLUH is expressed in the human intestine and intestinal macrophages, and CLUH protein expression is reduced only in the affected colonic region in patients with UC. (**A**) Hematoxylin and eosin staining was performed to check higher cellular infiltration in the colonic biopsy from patients with UC and control (non-UC). Original magnification, 20× and 40×, is shown. Data are representative of 3 donors in each group. (**B**) Immunohistochemistry was performed to visualize CLUH expression in the colonic biopsy from the control (non-UC) donors. Data are representative of 3 donors. (**C**) CLUH mRNA expression (*n* = 19, *n* = 20, *n* = 20 colon biopsies from the control, UC inactive region, UC active region) was checked. The level of each transcript is expressed after normalization to β-2-microglobulin. (**D**) Representative Western blot for CLUH expression with GAPDH as loading control is shown for 3 of 18 colon biopsies from non-UC control (N denotes non-UC, number denotes donor identification number), UC-unaffected region (P denotes patient-UC, number denotes donor identification number), and UC-affected region (P denotes patient-UC, number denotes donor identification number), along with a summary graph of densitometry in which samples are normalized to GAPDH (*n* = 18). (**E**) Purified macrophages from intestinal biopsy were used to assess CLUH protein expression with GAPDH as loading control (*n* = 6, non-UC donor). Mean ± SEM; ****P < 0.0001 as determined by 1-way ANOVA.

CLUH has previously been shown to be an RBP that regulates the translation and transport of NEMP to mitochondria, and CLUH-knockout epithelial cells display mitochondrial dysfunction and perinuclear clustering, with a functional complex II of the electron transport chain (24). Given colonic biopsy from patients with UC similarly shows a drastic mitochondriopathy with no change in complex II activity (10), we evaluated the contribution of CLUH in mediating mitochondrial function in patients with UC. Colonic biopsy sections showed enhanced cell infiltration in patients with UC versus the control groups (Figure 1A). In patients with UC, active disease and tissue immunohistochemistry studies revealed that CLUH was expressed in the infiltrating immune cells and the epithelial lining (Figure 1B). CLUH transcript level was similar between colonic biopsy from non-UC control groups and from obviously affected area and from apparently unaffected area from the same UC patients (Figure 1C). However, surprisingly, CLUH protein expression was drastically reduced specifically in the disease-affected region from patients with UC (Figure 1D and Supplemental Figure 1A; supplemental material available online with this article; https://doi.org/10.1172/jci.insight.161096DS1). Further experiments revealed that CLUH was highly expressed in purified human colonic macrophages (Figure 1E and Supplemental Figure 1B), and we chose macrophages as the cell type in which to investigate CLUH function. LPS, a bacterial endotoxin, which signals via host TLR4, is more abundant in UC colon, and TLR4 polymorphisms have been associated with UC susceptibility (28). Further, circulating endotoxin level is correlated with disease activity in IBD, and therapeutic reductions in endotoxin result in improvement of symptoms (29). We assumed an enhanced bacterial ligand availability in the affected colonic region might be responsible for the alteration of CLUH level and used multiple PRR-stimulated human macrophages as a model to understand the role of CLUH in immune regulation of IBD.

### Reduction in CLUH level is a prerequisite of cytokine secretion in PRR-activated human monocyte-derived macrophages.

**Figure 2:**
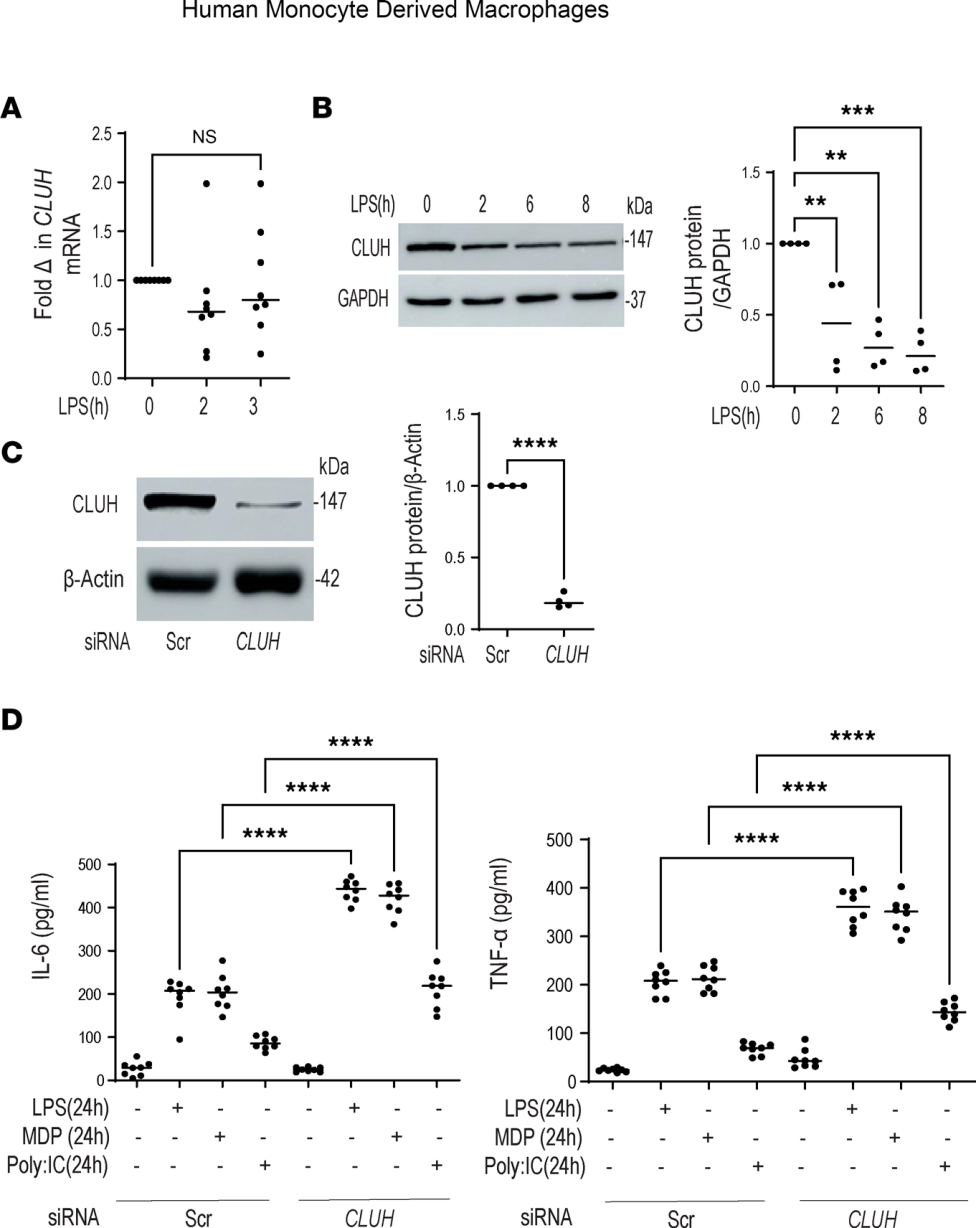
Reduction in CLUH level is a feature of PRR-activated human MDMs. Human MDMs were treated with 100 ng/mL LPS for the indicated times. (**A**) The level of *CLUH* transcript is shown after normalization to GAPDH (*n* = 8 donors). (**B**) CLUH protein expression by Western blot with GAPDH as a loading control along with a summary graph of a densitometry in which samples are normalized to GAPDH (*n* = 4 donors). (**C**) MDMs were transfected with scrambled and *CLUH* siRNA for 24 hours and assessed for transfection efficiency via CLUH protein expression along with a summarized densitometry (*n* = 4 donors, with similar result for an additional *n* = 4). (**D**) MDMs were transfected with scrambled and *CLUH* siRNA for 24 hours, then treated for 24 hours with 100 ng/mL LPS or 100 μg/mL bacterial muramyl dipeptide (MDP) or poly(I:C) 10 μg/mL; IL-6 and TNF-α secretion was measured from the cell supernatant (*n* = 8, with similar result observed in an additional *n* = 8 donors). Mean ± SEM; **P < 0.01; ***P < 0.001; ****P < 0.0001 determined by 2-tailed *t* test for **C** and 1-way ANOVA for the rest.

Human circulating monocytes enter the intestinal lamina propria and become tissue macrophages capable of producing a plethora of cytokines and dictating IBD pathogenesis (30, 31). In human monocyte-derived macrophages (MDMs), bacterial ligand LPS treatment showed no significant difference in CLUH mRNA level (Figure 2A); however, an early reduction in CLUH protein level was observed starting from 2 hours (Figure 2B). We assumed this reduction in CLUH level might be needed for LPS signaling and downstream pathways. In MDMs, CLUH knockdown with siRNA led to more than 90% reduction in CLUH protein level (Figure 2C), and these cells produced a significantly higher amount of proinflammatory cytokines like IL-6 and TNF-α compared with the cells transfected with scrambled siRNA, interestingly with no change in IL-1β and IL-10 secretion (Figure 2D and Supplemental Figure 2A). Similar results were observed with other TLR ligands, such as MDP and poly(I:C). Although CLUH-knockdown cells had higher cell death in the basal condition, there was no significant change in cell viability between control and CLUH-knockdown cells after LPS treatment (Supplemental Figure 2B). Additionally, during LPS+IFN-γ treatment, we observed a similar time-dependent decrease in the CLUH protein level (Supplemental Figure 2C). These data collectively demonstrate that reduction in CLUH is a prerequisite for PRR-induced inflammation in human macrophages and that CLUH acts as a negative regulator of proinflammatory cytokine secretion in activated macrophages.

### LPS treatment enhances CLUH-mediated ROS production, mtDNA release, and mitochondrial dysfunction.

**Figure 3:**
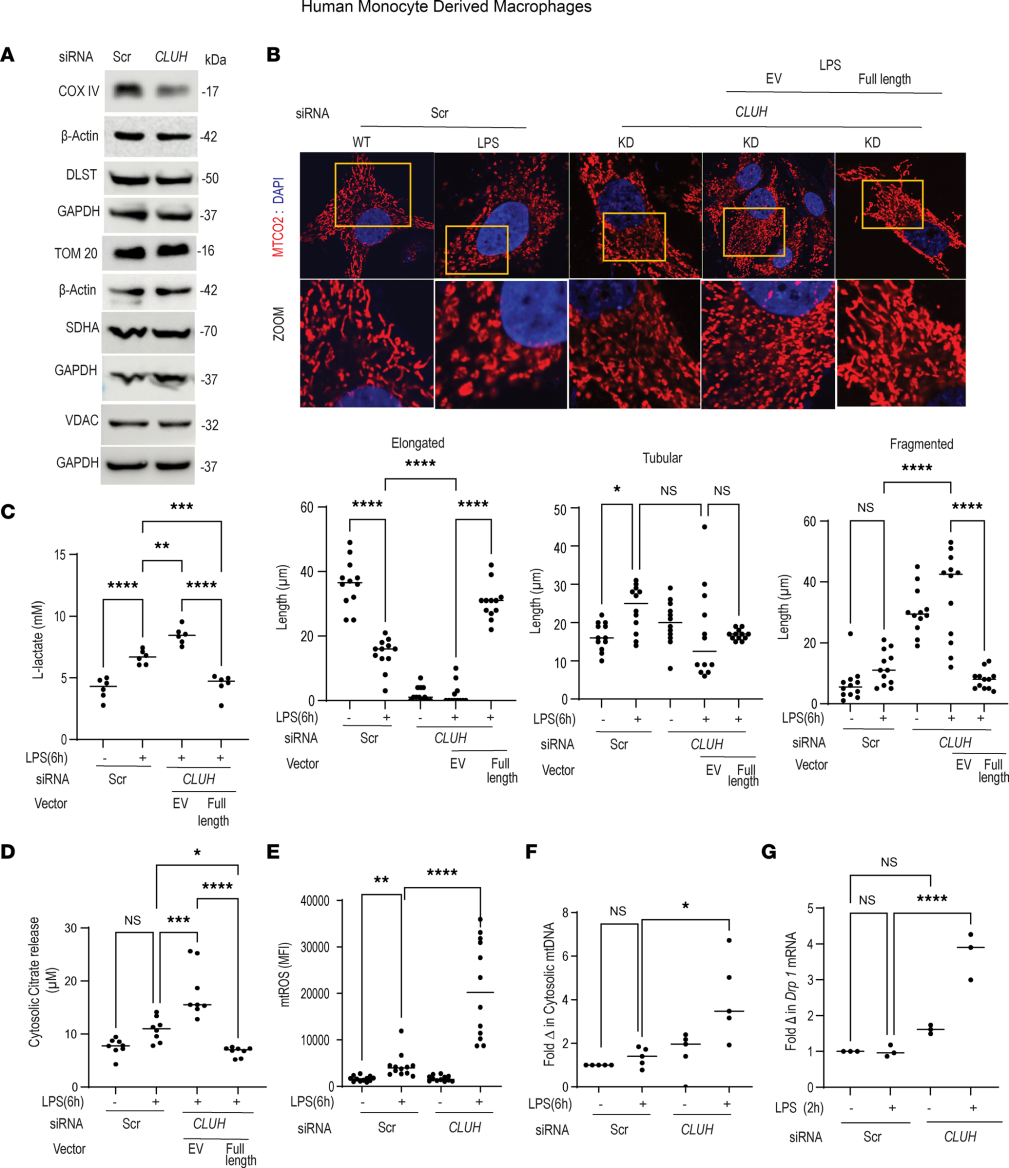
LPS treatment leads to CLUH-mediated early mitochondrial fission, mitoROS production, mtDNA release, and mitochondrial dysfunction. Human MDMs were transfected with scrambled or *CLUH* siRNA for 24 hours and treated with 100 ng/mL LPS for 6 hours and assessed for (**A**) mitochondrion-associated protein expression such as COX IV, DLST, TOM20, VDAC, and SDHA using GAPDH or β-Actin as a loading control (*n* = 1; with similar result observed in an additional *n* = 3 donors). Full-length CLUH plasmid (pEGFP-N1 vector) was also ectopically expressed in the CLUH-knockdown cells to rescue CLUH-knockdown condition. These cells were next treated with 100 ng/mL LPS for 6 hours and (**B**) stained with MTCO2 (red) as mitochondrial marker and DAPI (blue) to stain nucleus. Representative images of confocal microscopy and zoomed images are shown. Total original magnification, 63***×***. (**C**) Summarized number of elongated, tubular, and fragmented mitochondria from 1 experiment is shown. A total of 50–100 cells were acquired in 3 independent experiments. (**C**) l-Lactate activity and (**D**) mitochondrial citrate released into cytosol were estimated (pooled data from *n* = 6 and *n* = 8 samples, respectively, are shown). (**E**) Mitochondrial ROS (mitoROS) production (*n* = 12 donors); similar results were seen in an additional *n* = 9. (**F**) Mitochondrial DNA (mtDNA) release in the cytosol (*n* = 5; with similar results of an additional *n* = 3 donors). (**G**) mRNA expression of mitochondrial fission protein Drp1 after normalization with GAPDH (*n* = 3; with similar result for an additional *n* = 6 donors). Mean ± SEM; *P < 0.05; **P < 0.01; ***P < 0.001; ****P < 0.0001 as determined by 2-tailed *t* test for **E** and **F** and 1-way ANOVA for the rest of the figures. “EV” denotes empty vector.

We next wanted to understand how an RBP will alter inflammatory response in macrophages. Given the role of CLUH in mediating mitochondrial protein import in epithelial cells (24), we questioned if CLUH regulates mitochondrial dynamics in human macrophages. Unlike epithelial cells or hepatocytes, CLUH knockdown in human MDMs had little effect on the transport of mitochondrial proteins. We observed that after CLUH knockdown, there was marginal difference in the tested mitochondrial TCA cycle or electron transport chain component protein expression (Figure 3A and Supplemental Figure 3A). In epithelial cell lines CLUH knockout leads to perinuclear clustering of mitochondria (25). In contrast, after CLUH knockdown, human primary macrophages, which are more physiological, displayed a fragmented mitochondrial population. LPS treatment at early time points led to fragmentation of mitochondria, and after CLUH knockout there was significantly enhanced mitochondrial fragmentation, with a concomitant decrease in the elongated mitochondrial population (Figure 3B, zoomed images and summarized graphs). Ectopic CLUH overexpression (overexpression validated in Supplemental Figure 3B) in the knockdown cells reverted this phenomenon (Figure 3B, zoomed images and summarized graphs). After LPS stimulation, pseudo-hypoxic condition leading to mitochondrial dysfunction, TCA cycle break, and subsequent enhanced glycolysis is documented, leading to a “Warburg” situation (15). Similarly, epithelial cells with CLUH knockout are shown to have a dysfunctional mitochondrial pool and higher glycolysis (26). We speculated a reduction in CLUH level is a prerequisite for glycolytic reprogramming in the macrophages. As expected, data presented in Figure 3C clearly indicated that CLUH-knockdown cells after LPS treatment had higher glycolytic activity, with a pronounced TCA cycle intermediate citrate release (Figure 3D) from the mitochondria, than LPS-stimulated control cells. The glycolytic protein expression, however, was not altered (Supplemental Figure 3C). In addition to mitochondrial dynamics, we measured the mitoROS production, and after CLUH knockdown, human macrophages produced a significantly higher amount of mitoROS after TLR ligand stimulation (Figure 3E). We next measured the mtDNA release in the cytosol. As expected, after CLUH knockdown, macrophages had more mtDNA released in the cytosol, when compared with the control cells (Figure 3F). These data collectively demonstrate that CLUH is necessary to maintain the mitochondrial function in macrophages and that reduction in CLUH level may underlie the hyperfission phenomenon observed after LPS treatment. To gain insight into how CLUH might regulate mitochondrial size, we analyzed mitochondrial fission- and fusion-related transcripts. After 2 hours of LPS induction, cells with CLUH knockdown had higher DRP1 (Figure 3G). However, levels of mitochondrial fusion markers *Opa1* and *Mfn1* were not altered (Supplemental Figure 3D). We also observed that CLUH could directly bind to DRP1 in human MDMs (Supplemental Figure 3E) and probably sequesters it away from mitochondria to perform fission-related functions. These results explain the enhanced fission detected after CLUH knockdown and open up avenues for future research.

### Reduced mitophagy in CLUH-knockout human MDMs albeit smaller mitochondrial population.

**Figure 4:**
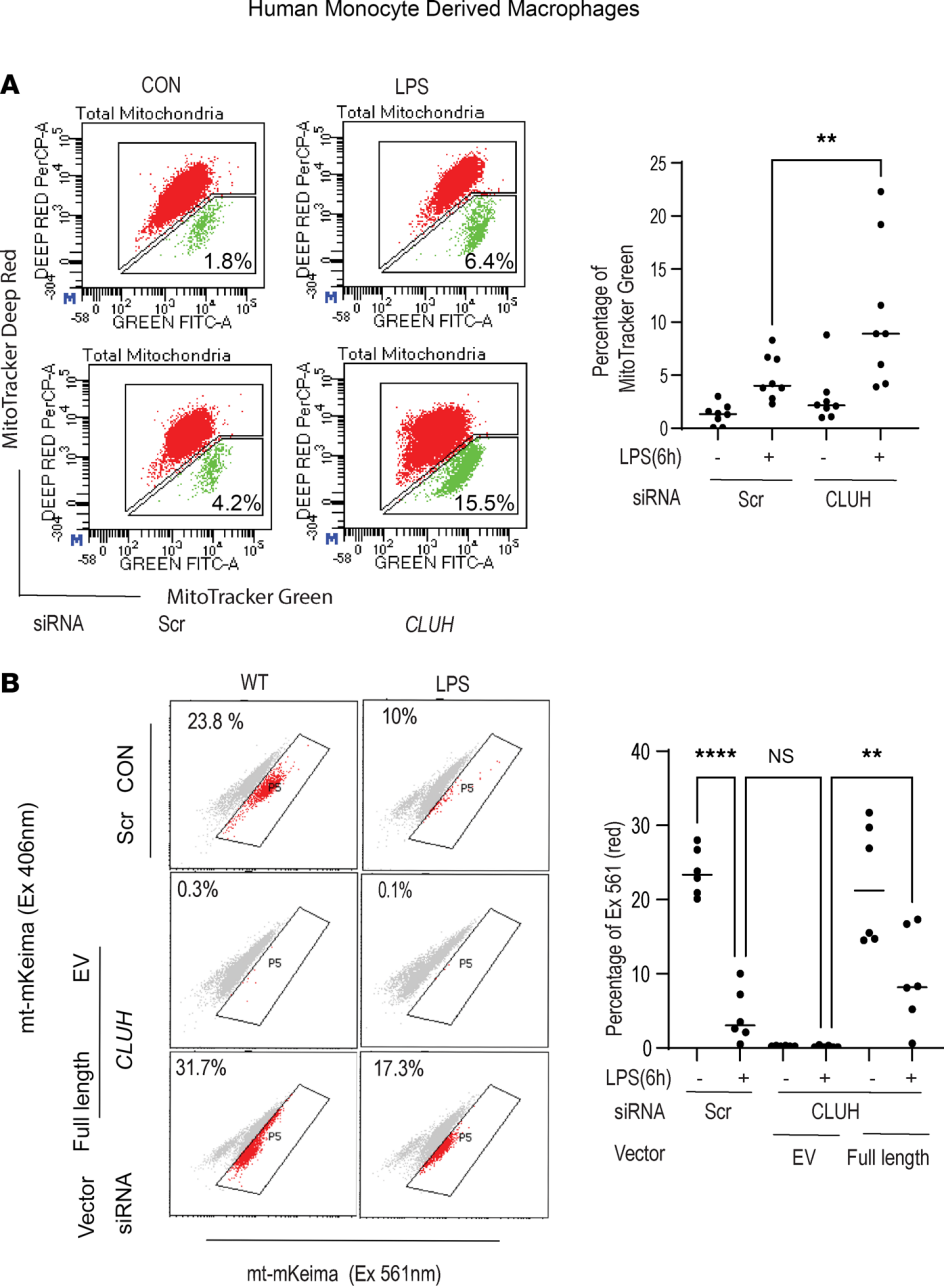
CLUH negatively regulates mitophagy in PRR-stimulated human macrophages. MDMs were transfected with scrambled or *CLUH* siRNA for 24 hours, treated with 100 ng/mL LPS for 6 hours, and (**A**) stained with MitoTracker Deep Red (for healthy mitochondria) and MitoTracker Green (for total mitochondria) for 30 minutes to assess mitochondrial dysfunction by flow cytometry. Representative and summarized flow cytometry with the mean fluorescence intensity (MFI) values are shown (pooled data from *n* = 8 donors are shown). (**B**) MDMs with CLUH-knockdown and full-length CLUH plasmid ectopically expressed in the CLUH-knockdown cells were transfected with mt-mKeima and assessed for mitophagy by flow cytometry at excitation 406 nm/excitation 561 nm. Representative and summarized flow cytometry with the MFI value shown (*n* = 6 samples); **B** is representative of 3 independent experiments. Mean ± SEM; **P < 0.01; ****P < 0.0001 as determined by 1-way ANOVA. “EV” denotes empty vector.

Mitochondrial fission and mitophagy are intrinsically linked as smaller dysfunctional mitochondria are targeted by lysosome-mediated clearance (32). The observation that CLUH knockout led to enhanced mitochondrial fission prompted us to study whether this smaller mitochondrial pool is subjected to mitophagy-mediated clearance. We first stimulated the human primary MDMs with LPS for 6 hours and stained them with MitoTracker Green and MitoTracker Deep Red. We noticed that dysfunctional mitochondria accumulated in the cells with reduced CLUH expression (Figure 4A). Mitochondrial relative position with lysosomes was not altered after CLUH knockdown (Supplemental Figure 4A). Mitophagy flux was next evaluated by flow cytometry using mt-mKeima Red, and after CLUH knockdown human MDMs displayed higher reduction in mitophagy after LPS stimulation when compared with the control cells (Figure 4B). When CLUH was ectopically expressed in cells with CLUH knockdown, mitophagy was rescued. Studies in a fly model have depicted that mitophagy adaptors PTEN-induce putative kinase 1 (PINK1) and Parkin (E3 ubiquitin protein ligase) bind to CLUH (33); however, in human macrophages these proteins were not functional (Supplemental Figure 3E) (34). We further explored expression of other mitophagy receptors such as NDP52, Beclin-1, and BNIP3 after CLUH knockdown. Data shown in Supplemental Figure 4B clearly displayed that none of the mitophagy markers tested were altered after CLUH knockdown. These results demonstrates that after CLUH knockdown, stressed and fissioned mitochondria do not undergo efficient clearance in the immune cells despite the fact that mitophagy receptor expression is not altered.

### Reduced mitophagy completion in CLUH-knockdown cells is via altered lysosomal activity.

**Figure 5:**
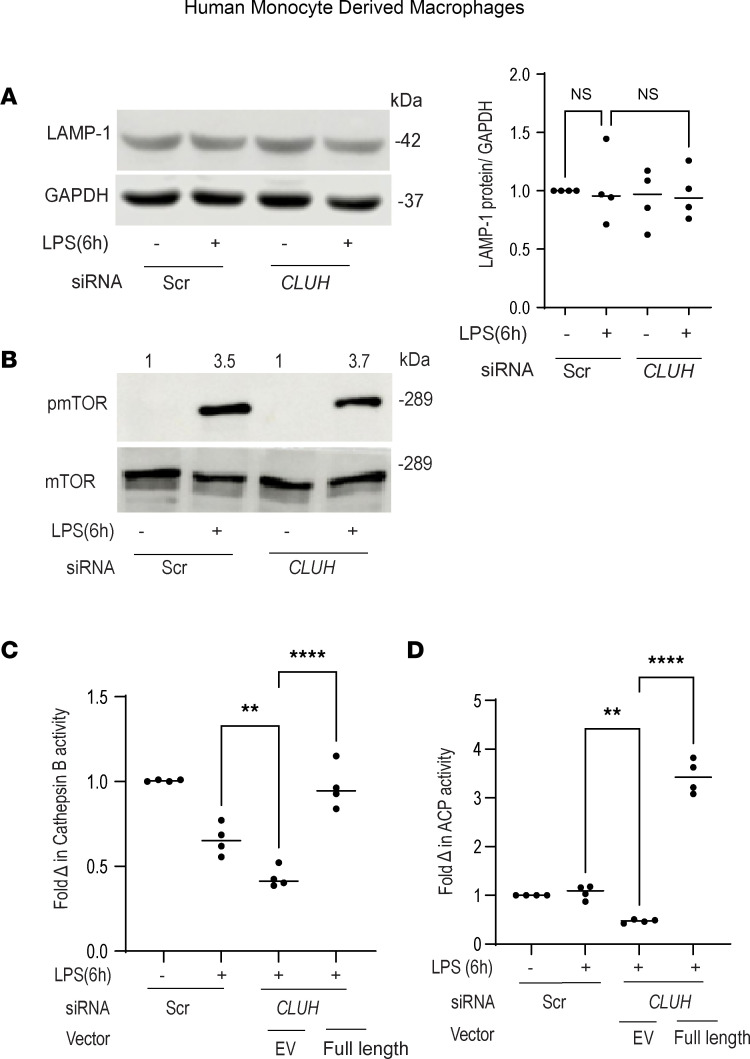
Reduced lysosomal activity is observed after CLUH knockdown. Human MDMs were transfected with scrambled or *CLUH* siRNA for 24 hours and treated with 100 ng/mL LPS for 6 hours. (**A**) LAMP1 expression by Western blot with GAPDH as a loading control along with a summary graph of a densitometry in which samples are normalized to GAPDH (*n* = 4). (**B**) Representative blot showing phosphorylated mTOR expression in *CLUH* siRNA–transfected MDMs treated with 100 ng/mL LPS for 6 hours along with a loading control mTOR (shown is 1 of 3 donors). For loading control, total mTOR was run in a different gel. Densitometry values are indicated above the band. Human MDMs were transfected with scrambled or *CLUH* siRNA for 24 hours or full-length CLUH plasmid ectopically expressed (pEGFP-N1 vector) in the CLUH-knockdown cells, then treated with 100 ng/mL LPS for 6 hours and assessed for (**C**) cathepsin B activity and (**D**) acid phosphatase activity. **C** and **D** are representative of 2 independent experiments. Mean ± SEM; **P < 0.01; ****P < 0.0001 as determined by 1-way ANOVA. “EV” denotes empty vector.

Enhanced mitoROS and DRP1-mediated mitochondrial fragmentation have been shown to induce lysosomal dysfunction (35–37). Hence, we next evaluated if altered mitophagy after CLUH knockdown was due to a difference in the lysosomal function. Lysosomal mass as measured by lysosomal associated membrane protein 1 (LAMP1) immunoblotting was similar between the controls and cells with CLUH knockdown (Figure 5A). In hepatocytes, CLUH regulates mitophagy by maintaining mTOR complex 1 (mTORC1) activity, and mTOR can directly regulate lysosomal activity (25). However, in our setup with human primary macrophages, CLUH had no effect on mTOR activation (Figure 5B). This ambiguity can be explained by the cell-intrinsic role of CLUH, and possibly CLUH functions in an altogether different manner in macrophages, which are clinically more relevant in deciding disease pathology. Intriguingly, after CLUH knockdown, acid-dependent lysosomal proteolytic activity level was altered. Cells with reduced CLUH level had significantly low cathepsin B activity (Figure 5C), and lysosomal acid phosphatase level (Figure 5D) and CLUH overexpression rescued this defect. Lysosomal inhibitors would have no further effect on mitophagy if indeed the mechanism involves lysosomal degradative insufficiency, and our next experiment provided functional evidence supporting this idea. Lysosomal inhibitor bafilomycin A1 treatment in human MDMs (Supplemental Figure 5A) exhibited no significant added effect on mitophagy reduction in CLUH-depleted cells.

To explore whether this lysosome insufficiency has any impact on other forms of autophagy, we measured autophagy level in the presence of cytoID, a dye staining autophagic vacuoles. CLUH knockdown had a mild effect on autophagy, and no added effect of lysosomal inhibitor chloroquine on the autophagic flux in CLUH-knockdown cells was observed (Supplemental Figure 5B). LC3 is a mitophagy adaptor protein, and cells with CLUH knockdown had slightly reduced LC3II level (Supplemental Figure 5C). These data further indicate that LC3II level was not increased in the presence of bafilomycin A1 in the cells with CLUH knockdown. This suggests that decreased formation rather than increased degradation of LC3 was the underlying reason for the observed reduction of LC3 in the CLUH-depleted cells. Together, these observations clearly point to a major effect of degradative insufficiency of lysosomes and impaired degradative stage of mitophagy in CLUH-depleted cells.

### CLUH alters cytokine production by maintaining mitochondrial function in human MDMs.

**Figure 6:**
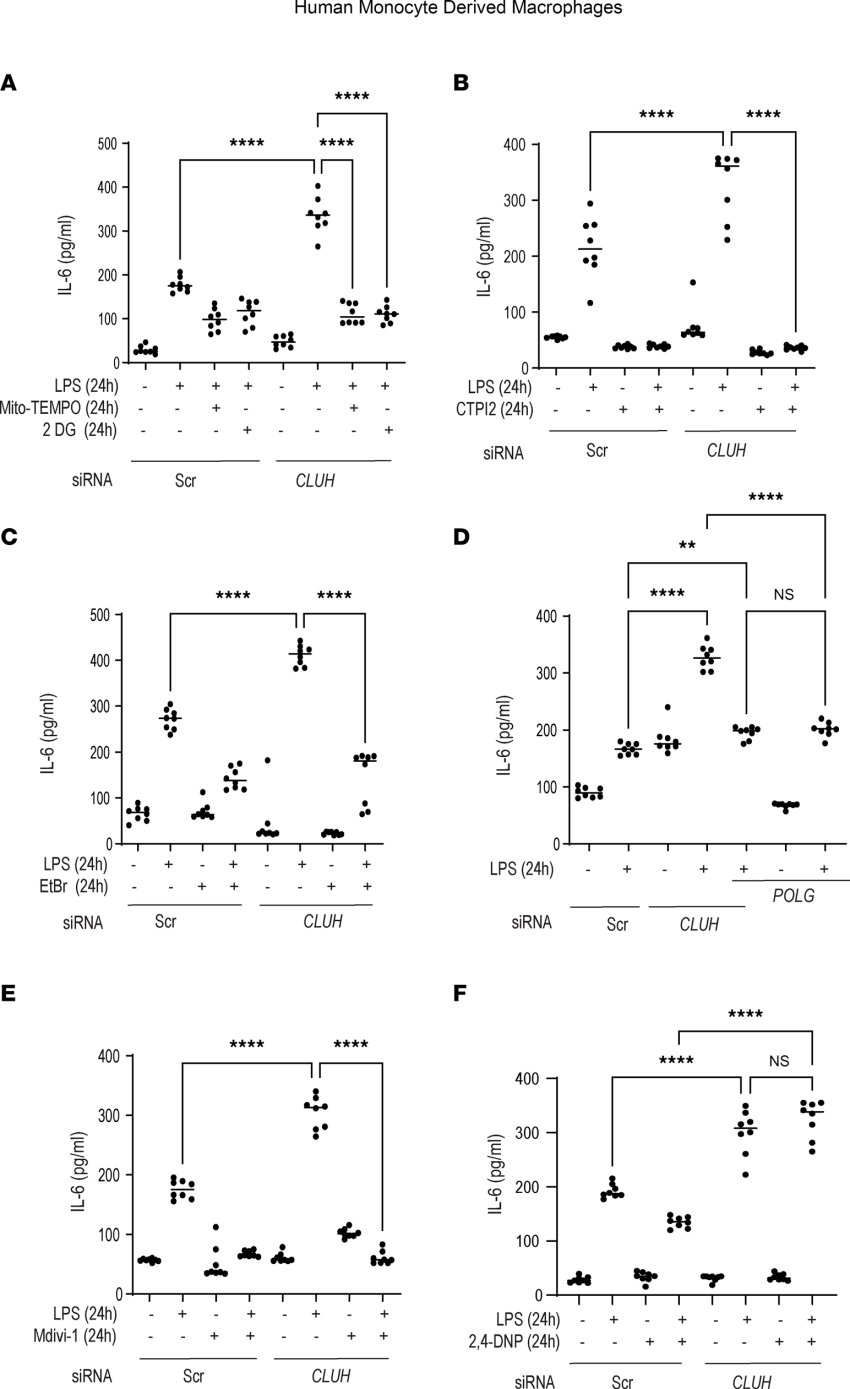
CLUH regulates cytokine production downstream of LPS by maintaining mitochondrial function and dynamics in human MDMs. Human MDMs were transfected with scrambled or *CLUH* siRNA for 24 hours and treated with 100 ng/mL LPS for 24 hours. (**A**) IL-6 secretion after treatment with or without Mito-TEMPO (1 μM, to inhibit mitoROS production) or 2-deoxy-d-glucose (5 mM, to inhibit glycolysis). (**B**) IL-6 secretion after treatment with or without CTPI-2 (1 mM, to inhibit mitochondrial citrate export). (**C**) IL-6 secretion after treatment with or without EtBr (50 ng/mL to reduce mtDNA quantity). (**D**) IL-6 secretion after transfection with or without *POLG* siRNA. (**E**) IL-6 secretion after treatment with or without Mdivi1 (50 μM, to inhibit mitochondrial fission). Sources of reagents are in Supplemental Table 3. (**F**) IL-6 secretion after treatment with or without 2,4 DNP (50 μM, to activate mitophagy). (Pooled data from *n* = 8 donors.) Mean ± SEM; **P < 0.01; ****P < 0.0001 as determined by 1-way ANOVA.

So far our results reveal that CLUH regulates mitochondrial dynamics and proinflammatory cytokine production after TLR stimulation and controls the commitment of macrophages toward an inflammatory phenotype. There exists a close relationship between mitophagy and cytokine production (38). To better understand the reason behind enhanced proinflammatory cytokine production in CLUH-depleted macrophages, we used 2-deoxy-d-glucose, inhibitor of glycolysis, and Mito-TEMPO, inhibitor of mitoROS, and observed that the cytokine secretion was similar to the control cells (Figure 6A) (36). Mitochondrial citrate release in the cytosol was more prominent after LPS stimulation in the cells with CLUH knockdown. This citrate may provide an alternative mechanism to explain the proinflammatory niche when CLUH is dysfunctional. We stimulated the cells with LPS in the presence or absence of CTPI-2, a specific inhibitor of mitochondrial citrate secretion, and noted that IL-6 production was similar between the control and CLUH-knockdown cells (Figure 6B). If the enhanced mtDNA released in the cytosol is responsible for the higher proinflammatory cytokine production downstream of CLUH, these effects should be abrogated if we use ethidium bromide (EtBr), which reduces mtDNA and reduces polymerase-G (POLG), which is essential for mtDNA synthesis. As expected, data shown in Figure 6, C and D verified that mtDNA was also partly responsible for the enhanced cytokine production in the LPS-stimulated macrophages after CLUH knockdown. In order to verify if the enhanced mitochondrial fission and reduced mitophagy were also responsible for the altered cytokine production in CLUH-depleted cells, we used Mdivi1, a specific mitochondrial fission inhibitor. LPS-stimulated cytokine secretion after CLUH knockdown was similar to the control cells in presence of Mdivi1 (Figure 6E). 2,4 DNP, a mitophagy initiator, was next used in order to restore the mitophagy deficiency in the CLUH-depleted cells, and it could not reduce the enhanced cytokine secretion after CLUH knockdown (Figure 6F). All the inhibitors and siRNA used in these experiments were validated to check that they inhibit their intended targets (Supplemental Figure 6, A–G). These results verify that CLUH is necessary for keeping the cytokine production in check in PRR-stimulated macrophages. After CLUH reduction, the enhanced mitoROS, mtDNA, and citrate secretion and smaller mitochondrial pool (not undergoing mitophagy) contribute together toward higher proinflammatory activation in human macrophages.

### Cluh knockdown in C. elegans similarly enhances mitochondrial fission.

**Figure 7:**
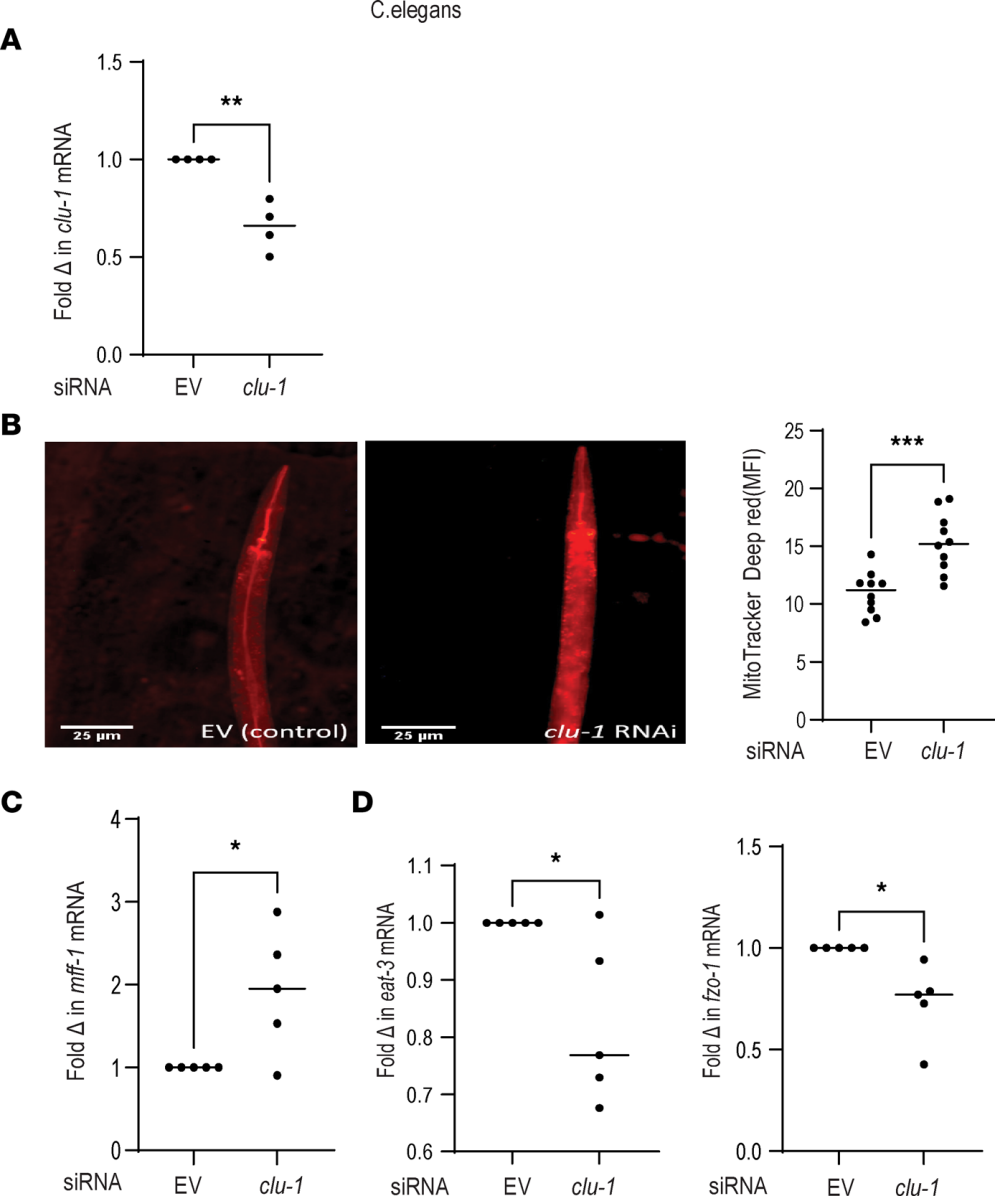
CLUH knockdown in *C*. *elegans* similarly enhances mitochondrial fission. N2 strain of *C*. *elegans* was fed with HT115 bacteria carrying an empty vector or siRNA against *Clu-1* for 24 hours. (**A**) Transcript level of clu-1 after normalization to β-Actin (pooled data from *n* = 4 samples are shown). (**B**) Total mitochondrial content in the form of red fluorescent dots as observed under fluorescence microscope using MitoTracker dye (100 nM) along with graphical representation of relative change in fluorescence intensity (10 fields for each condition). (**C**) mRNA expression of mitochondrial fusion marker *mff1*. (**D**) mRNA expression of fission markers *eat3* and *fzo1* normalized to *β-Actin* (pooled data from *n* = 5 samples are shown). Mean ± SEM; *P < 0.05; **P < 0.01; ***P < 0.001 as determined by 2-tailed *t* test. “EV” denotes empty vector.

Next, we wanted to validate if the mitochondrial dynamics alteration by CLUH is translated in vivo, and we employed a *C*. *elegans* N2 (Bristol) model for conducting the next set of experiments. *Clu1* gene silencing was carried out by employing the feeding protocol, and we observed significant knockdown efficiency in the CLU1 (homolog of human CLUH) expression (Figure 7A). Knockdown of *clu-1* had no effect on synchronized stages of development (embryos**→**L1**→**L2**→**L3**→**L4) or the body size of young adult or L4-staged worms when compared to the control worms (Supplemental Figure 7, A and B). We then explored the mitochondrial dynamics in these worms, and data shown in Figure 7B indicate that Clu1 knockdown translated into a pronounced increase in the mitochondrial density. The above phenomenon was attributed to a higher level of mitochondrial fission inducing gene (*mff1*, Figure 7C) and a reduced level of fusion-related genes like *eat3* and *fzo1* (Figure 7D) in the worms after Clu1 knockdown. For better understanding the reason behind the observed enhancement of mitochondrial number in these worms in the absence of clu-1, we explored mitochondrial biogenesis markers skn-1, atp-5, hmg5, and gas-1 and mitophagy markers pdr-1 and pink-1. Data suggested that clu-1 knockdown had no effect on mitochondrial biogenesis and/or mitophagy markers (Supplemental Figure 7, C and D). Collectively, these results indicate that just like human MDMs, clu-1 knockdown leads to enhanced mitochondrial fission pathways.

### Cluh knockdown in mice exacerbates disease pathology in the dextran sodium sulfate–induced colitis model.

**Figure 8:**
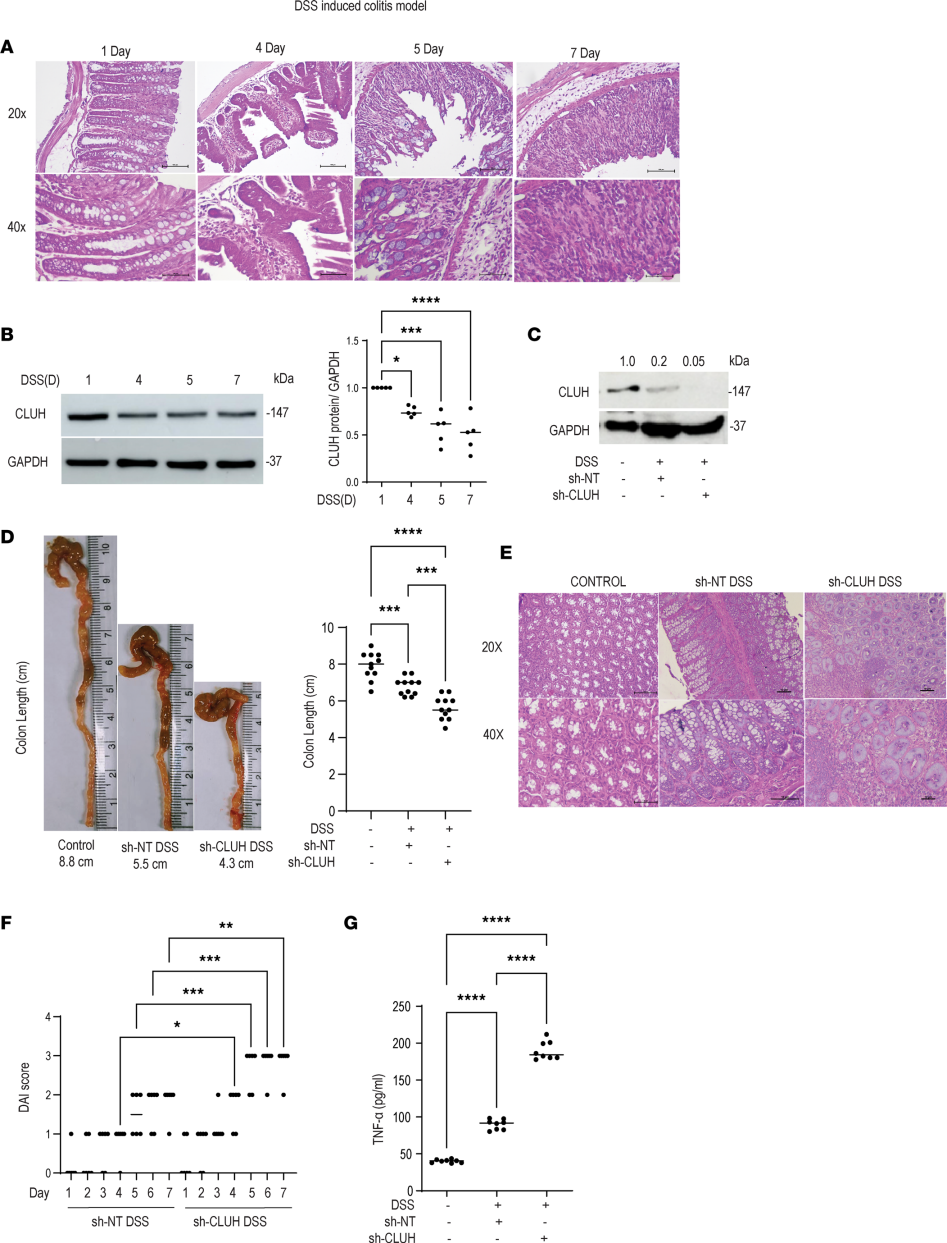
*Cluh* knockdown in mice exacerbates disease pathology in a DSS-induced colitis model. C57BL/6 male mice (7 weeks old) were fed DSS (2.5%) in drinking water (*n* = 5) for 7 days and assessed for (**A**) hematoxylin and eosin (H&E) staining of colon tissue in a day-dependent manner (1 day, 4 days, 5 days, 7 days) using Leica DMi-5000 microscope, and 20× and 40× magnified representative images are shown. (**B**) CLUH expression in colon tissue in a day-dependent manner (1 day, 4 days, 5 days, 7 days) by Western blot with GAPDH as a loading control and summarized densitometry from 5 animals. *Cluh* shRNA or nontargeted shRNA expressing lentivirus was administered via i.p. route (5 × 10^6^ particles/mouse on 7 and 1 day before DSS administration) to reduce the expression of CLUH and checked for (**C**) CLUH knockdown confirmation by Western blot (1 of 4 replicates) with GAPDH as a loading control. Densitometry values are indicated above the band. (**D**) Representative colon length with the summary graph from pooled data from 11 animals from 2 independent experiments in each group. (**E**) H&E staining of colon tissue using Leica DMi-5000 microscope: 20× and 40× magnified representative images are shown. (**F**) DAI score is shown for the animals. (**G**) TNF-α production from colon tissue lysates (8 animals in each group). Mean ± SEM; *P < 0.05; **P < 0.01; ***P < 0.001; ****P < 0.0001 as determined by 1-way ANOVA.

After validating the in vivo role of CLUH in maintaining mitochondrial dynamics in the worm model, we employed a murine mucosal inflammatory model of UC. The dextran sodium sulfate–induced (DSS-induced) colitis model (which mimics UC pathology) has been widely used during IBD drug discovery and immune pathway studies, specifically in understanding cytokine secretion from macrophages after TLR stimulation (39, 40). Interestingly, mitochondrial pathways were previously shown to be altered in the DSS-induced colitis model (5, 9, 41), and we used this model to understand in vivo function of CLUH. Colon sections from animals treated with DSS for 1, 4, 5, and 7 days were stained with H&E, and there was a time-dependent increase (starting from the fourth day) in the inflammatory cell migration and associated disease scores in the colon (Figure 8A and Supplemental Figure 8A). Like in the patients with UC, DSS treatment also led to a reduced CLUH level (Figure 8B), and this decrease matched with the inflammation time points as observed in Figure 8A. CLUH-knockout animals are embryonic lethal (26); hence, we utilized lentivirus-mediated knockdown of CLUH using specific shRNA in the DSS-induced colitis model. We could effectively reduce the CLUH level in the colon after i.p. injection of the lentivirus containing CLUH shRNA (Figure 8C). After 7 days of DSS treatment the colon sections from CLUH shRNA–injected animals displayed significantly reduced body weight (Supplemental Figure 8B) and smaller colon length (Figure 8D). Further, higher inflammatory cell migration, inflammation-associated scores (Figure 8E and Supplemental Figure 8C), and enhanced disease activity index (DAI) (Figure 8F) were observed after CLUH knockdown when compared with the nontargeting shRNA–injected animals. The body weight and the DAI scores clearly showed that starting from the fourth day of DSS treatment (time point where we saw a reduction at CLUH level in Figure 8B), CLUH knockdown enhanced the disease progression when compared with the control mice. Finally, we quantified the IL-6 production from the colon tissues, and the data mirrored our in vitro observations. Data shown in Figure 8G demonstrate that cytokine production was controlled by CLUH in the animal model of colitis as well and that CLUH knockdown after DSS treatment showed a 2-fold increase in TNF-α secretion.

### LPS-induced CLUH degradation is mediated via ubiquitination and sumoylation at residue Lys1257.

**Figure 9:**
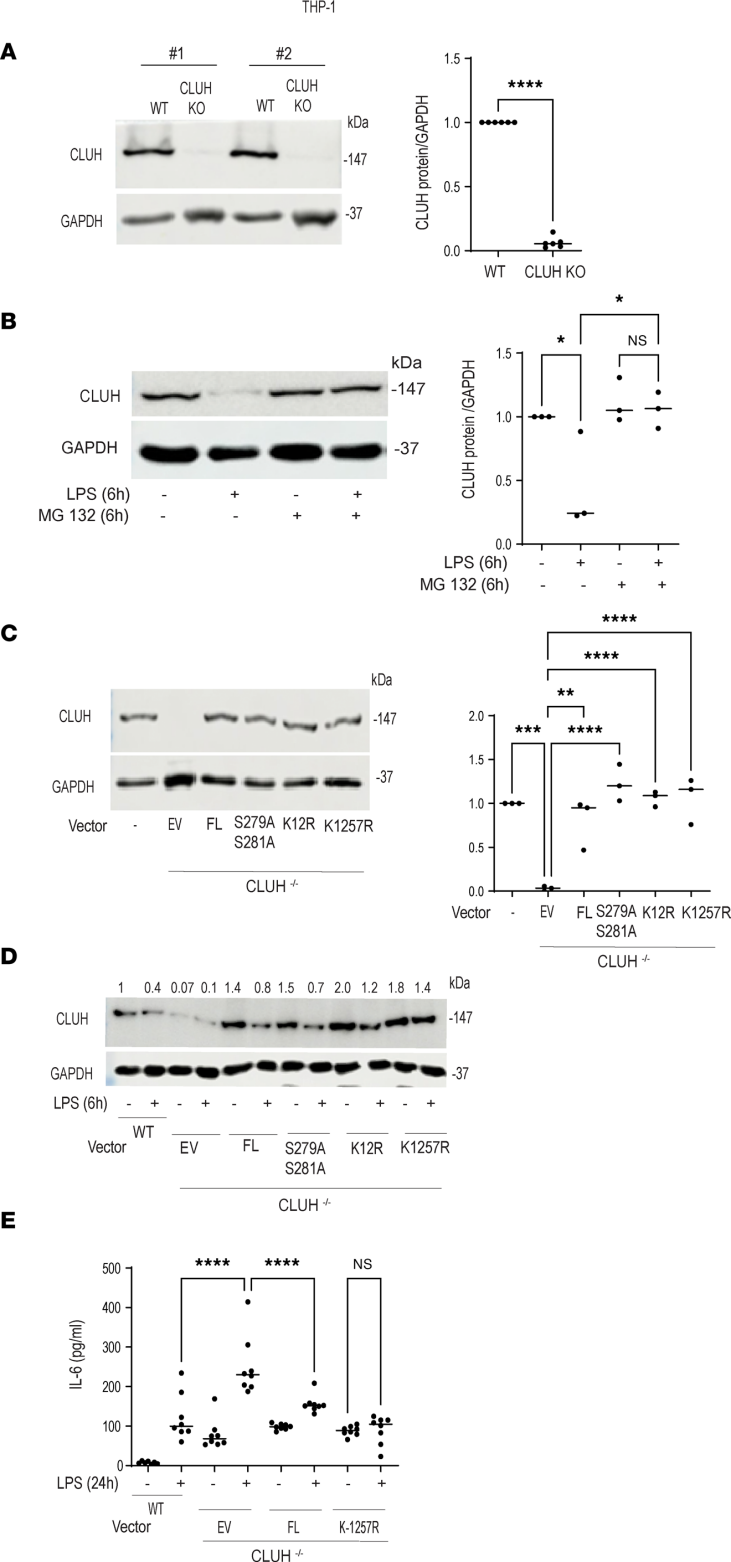
LPS-induced CLUH degradation is mediated via sumoylation/ubiquitination at residue Lys1257. (**A**) Confirmation of CRISPR-mediated CLUH knockout in THP1 cells by Western blot in which GAPDH is used as loading control (2 of 6 technical replicates shown) along with summary graph of densitometry from 6 replicates. (**B**) WT THP1 cells were pretreated with proteasome inhibitor (MG132, 50 μM) for 6 hours and treated with 200 ng/mL LPS for 6 hours. CLUH protein level was checked by Western blot in which GAPDH is used as loading control (1 of 3 replicates shown) along with summary graph of densitometry from 3 replicates. (**C**) THP1 CLUH-knockout (CLUH-KO) cells were transfected with *CLUH* full length (FL) or *CLUH* S279,281A; *CLUH* K-12; and *CLUH* K1257 mutants (pEGFP-N1 vector) and Western blot for CLUH expression GAPDH as a loading control in 1 of 3 replicates, along with summary graph of densitometry from 3 replicates. WT THP1 cells are used as control. (**D**) THP1 CLUH-KO cells were transfected with *CLUH* FL or *CLUH* S279,281A; *CLUH* K-12; and *CLUH* K1257 mutants and treated with 200 ng/mL LPS for 6 hours. Western blot for CLUH expression using GAPDH as a loading control in 1 of 4 replicates. Densitometry values are indicated above the band. (**E**) IL-6 production from the supernatants (8 replicates from each group) from THP1 CLUH-KO cells after transfection with *CLUH* FL or *CLUH* K1257 mutants and treated with 200 ng/mL LPS for 24 hours. WT THP1 cells are used as control. Mean ± SEM; *P < 0.05; **P < 0.01; ***P < 0.001; ****P < 0.0001 as determined by 2-tailed *t* test (**A**) and 1-way ANOVA for the rest. “EV” denotes empty vector.

High LPS exposure is documented in the colon of human patients with UC and in the DSS-treated animals. In both these conditions, we observed reduced CLUH expression. This led us to investigate how PRR stimulation at early time points might lead to CLUH protein (not RNA) downregulation. We constructed a CRISPR-mediated CLUH-knockout human monocyte THP1 cell line (Figure 9A) and used this cell line to understand the molecular mechanism of CLUH degradation downstream of PRR signaling. First, we observed that in the THP1 cells, CLUH level was reduced after LPS stimulation like in the human primary macrophages. However, treating the LPS-stimulated cells with MG132, a cell-permeable proteasome inhibitor, completely restored the CLUH protein level to that of the unstimulated cells, suggesting involvement of the proteasome in this degradation pathway (Figure 9B). Proteasome-mediated degradation occurs after sumoylation and/or ubiquitination. Sumoylation in host proteins has been shown to be involved in mitophagy (42); hence, we analyzed the CLUH sequence for potential sites that can be sumoylated and/or ubiquitinated and found 2 sites at Lys12 and Lys1257 with high sumoylation probability scores (Supplemental Figure 9A). In addition, phosphorylation of proteins is also associated with degradation, and 2 potential phosphorylation sites at Ser279 and Ser281 were selected based on their sequence homology between humans and mice to evaluate their contribution in the LPS-mediated degradation pathway (Supplemental Figure 9B). We mutated the lysine residues to arginine and serine residues to alanine and transfected the mutant plasmids in the CLUH-knockout cells. Protein expression was comparable between the WT CLUH plasmid and all the mutants in the transfected cells (Figure 9C). Lys12Arg and Ser279,281Ala double mutants showed similar reduction in CLUH level as the full-length WT CLUH plasmid. Importantly, the Lys1257Arg mutant did not show LPS-mediated degradation, and this result suggests that this particular residue is crucial in the proteasome-mediated degradation pathway of CLUH after PRR stimulation. This Lys1257 site also had a ubiquitin-binding site, and probably a consort of sumoylation and ubiquitination predisposes CLUH for degradation. We cannot obviously exclude the participation of other residues; however, results from Figure 9D showed Lys1257 was a critical residue in CLUH that was targeted by sumoylation and degradation. It is noteworthy that LPS-induced IL-6 production was augmented in the CLUH-knockout THP1 cells (Figure 9E), in line with CLUH being a negative regulator of inflammation. This enhanced cytokine production was mitigated after transfection with CLUH full-length plasmid in these knockout cells. In contrast, Lys1257Arg mutant, when transfected in the CLUH-knockout cells, did not show enhanced IL-6 production, clearly pointing to the fact that CLUH degradation at Lys1257 is necessary for LPS-induced cytokine secretion.

### Patients with UC have higher mitochondrial fission and reduced mitophagy-associated gene expression from their colonic tissues.

**Figure 10:**
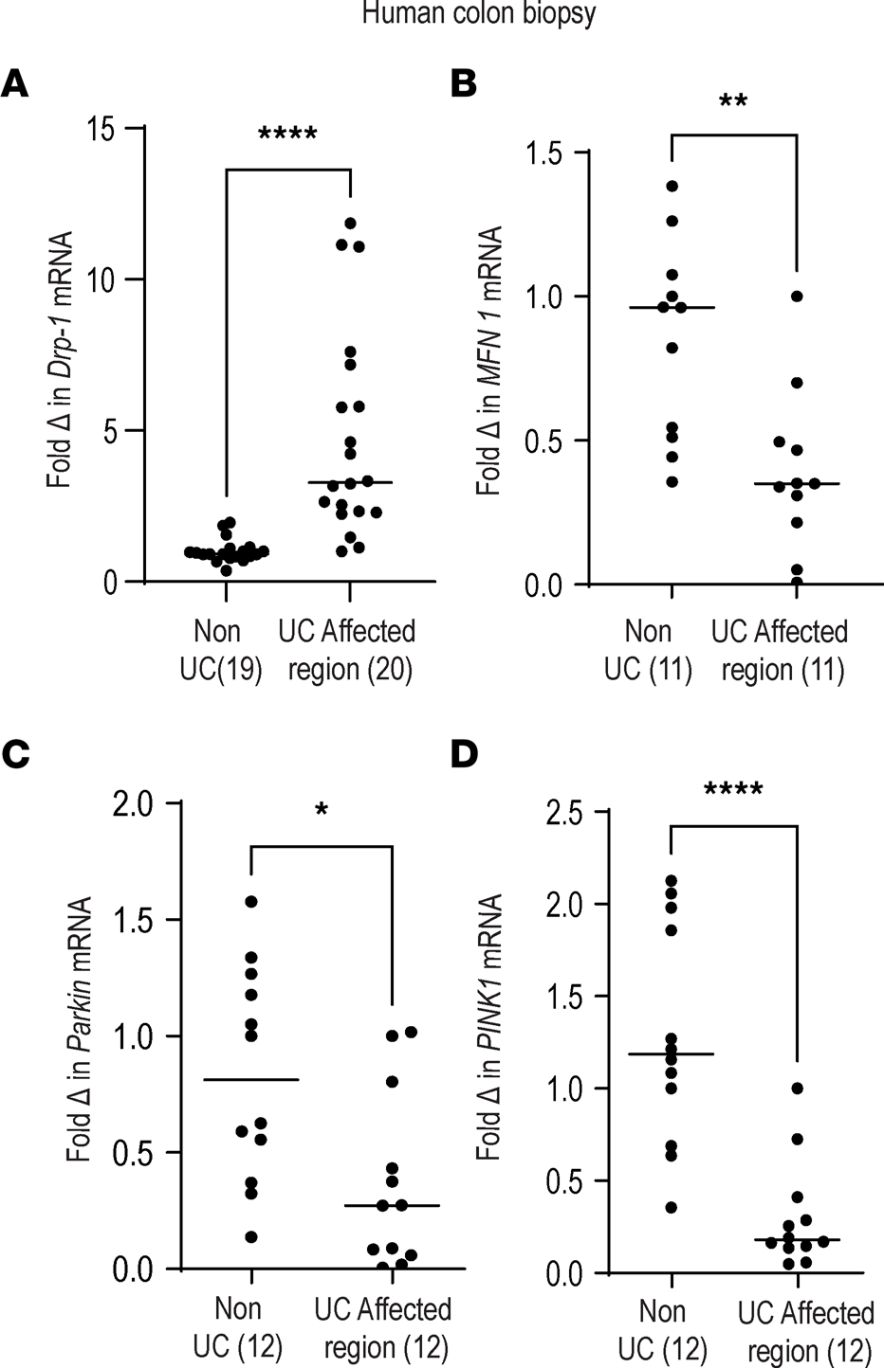
Patients with UC have higher mitochondrial fission and reduced mitophagy-associated transcripts in colonic tissues. Human colonic biopsy tissue of non-UC control and UC patients (from affected areas) were assessed for (**A**) mRNA expression of mitochondrial fission marker *Drp1* (*n* = 19 non-UC, *n* = 20 UC), (**B**) fusion marker *Mfn1* (*n* = 11 non-UC, *n* = 11 UC), (**C**) mitophagy pathway marker *Parkin* (*n* = 12 non-UC, *n* = 12 UC), and (**D**) mitophagy pathway marker *PINK1* (*n* = 12 non-UC, *n* = 12 UC). The level of each transcript is expressed after normalization to β-2-microglobulin. Mean ± SEM, *P < 0.05; **P < 0.01; ****P < 0.0001 as determined by 2-tailed *t* test analysis.

We initially showed that colonic biopsies from patients with UC had a significant reduction in their CLUH level. We also documented that in human macrophages, CLUH knockout resulted in enhanced mitochondrial fission, mitophagy inhibition, and lysosomal dysfunction. Finally, we sought to correlate the clinical relevance of these in vitro findings in patients with UC of our cohort. In the patients with UC, indeed we observed an increased expression of DRP1 and reduced MFN1 level from their colonic biopsy specimens, indicating a smaller mitochondrial pool (Figure 10, A and B). Further, mitophagy-associated proteins Parkin and PINK1 were also significantly underexpressed in patients with UC (Figure 10, C and D). Confocal microscopy from the biopsy sections showed a trend toward higher mitochondrial fluorescence intensity in patients with UC (Supplemental Figure 10A). Finally, by transmission electron microscopy (TEM) (43), we observed higher fissioned mitochondria in the affected colonic region from the UC than the non-UC group (Supplemental Figure 10B). Collectively, these results support the role of CLUH specifically in IBD pathogenesis and inflammation in general. In summary, a reduction in the CLUH level in the affected region of the colon facilitates the mitochondrial fragmentation, reduced mitophagy, and higher inflammation observed in patients with UC (Supplemental Figure 10C).

## Discussion

Our findings reveal that CLUH, a cellular RBP, regulates mitochondrial function in myeloid cells, specifically after PRR stimulation. CLUH maintains mitochondrial dynamics and allows mitophagy to happen in macrophages. After LPS stimulation, CLUH level was reduced in the cell, and this reduction is necessary for efficient proinflammatory cytokine secretion and macrophage polarization to M1 phenotype. After LPS stimulation, mitochondrial function was disturbed, cellular glycolysis was, enhanced and we observed CLUH had a crucial role in this LPS-induced Warburg effect as well. The translational values for these findings were documented in the pathogenesis of UC. The affected region from the colonic tissues of patients with UC showed a drastic underexpression of CLUH and associated higher inflammation and low mitophagic pathways. For the first time to our knowledge, we identify CLUH as a master suppressor of innate immune response in the PRR-stimulated macrophages and in the intestinal mucosa (Supplemental Figure 10C).

RBPs are essential in maintaining the stability, localization, and translational efficiency of cellular RNA, and their malfunction underlies the origin of many diseases (44). CLUH is an RBP that has been shown to specifically bind to transcripts encoding mitochondrial proteins and help in their stability, translation, and efficient transport to mitochondria in epithelial cells, hepatocytes, and adipocytes (23, 24, 26). We investigated the role of CLUH in immune cells and found a marginal effect of CLUH in regulating transport of mitochondrial proteins in macrophages, and the clustering phenomenon was absent as well in the absence of CLUH. Interestingly, CLUH was found to bind to DRP1 and regulate its transcription (CLUH knockdown enhanced DRP1 only after early LPS treatment) to dictate mitochondrial morphology in human primary macrophages. We believe that in the absence of CLUH/LPS treatment, a retrograde signaling from the dysfunctional mitochondria allows more drp1 transcription to enhance mitochondrial fission and mitophagy. However, after CLUH knockdown/LPS treatment higher mitoROS-mediated lysosomal dysfunction precluded mitophagy of these fissioned mitochondria. We believe that these functions of CLUH are more effective in the specific immune cells, like human MDMs, and more prominent after PRR stimulation.

Mitochondrial function and morphology could dictate immune outcome by various means and have been well documented in human diseases (4, 45). Indeed, the role of CLUH in regulating mitochondrial dynamics was pronounced after PRR stimulation. LPS or other PRR stimulation led to mitochondrial dysfunction and cellular energy synthesis routed toward enhanced glycolytic pathway, and we identified a role of CLUH in this PRR-induced Warburg effect. CLUH level was reduced after PRR stimulation, and this led to a dysfunctional and smaller mitochondrial pool in the immune cells, which made each cell depend on glycolysis for energy needs. This in turn enhanced the cytokine secretion from the stimulated macrophages and TCA cycle intermediates, like citrate, to be released in the cytosol. Mitochondrial fission is associated with higher glycolysis, and M1 macrophages rely on aerobic glycolysis for energy production (46, 47). The complex interplay between the M1 and M2 macrophages decides the inflammatory and profibrotic, proantigenic, and wound-healing features and plays a crucial role in UC pathogenesis (48). However, single-cell sequencing has shown that both M1 and M2 phenotype can be displayed by the same cells, and M2 macrophages can switch to M1 phenotype (49–51). We observed that CLUH level was similarly reduced during macrophage polarization, indicating a possible role of CLUH in this regard, and future studies should explore the role of CLUH in M1 and M2 outcomes.

A fissioned and dysfunctional mitochondrial pool undergoes mitophagy-mediated degradation in the quiescent cells or during starvation (52). Surprisingly, our data demonstrated that after LPS stimulation, these mitochondria did not undergo efficient clearance in the CLUH-knockout cells. LPS treatment has earlier been shown to induce mitochondrial fission, enhance mitoROS production, and reduce mitophagy (19, 20), and CLUH might be the missing link between these 2 contrasting phenomena. CLUH was previously shown to regulate starvation-induced mitophagy via inhibiting mTORC1 pathway in murine hepatocytes (25). However, this is not the case in human primary macrophages as we did not observe any difference in mTOR pathway after CLUH knockdown. A potential explanation for the defective mitophagic flux came after closely looking at the lysosomal pathways. Loss of mitochondrial function and higher mitoROS have been shown to impair lysosomal functions during diabetic conditions (35) and neurodegenerative disorders (36). Abnormal mitochondria perturb lysosomal function in the T cells and exacerbate DSS-induced inflammation in mice (53). Here we provide evidence that lysosomal degradation pathways are altered after CLUH knockdown, which suggests evidence of a dysfunctional mitochondria-lysosome crosstalk and inhibition of the degradation step of mitophagy.

Recent studies support mitochondrial dysfunction in patients with IBD (54, 55), and increased intestinal inflammation is associated with reduced mitochondrial activity in the animal model of colitis (56). Low ATP production and enhanced mtDNA release in the blood have been further observed in patients with UC (57). Autophagy and mitophagy can negatively regulate cytokine production (3, 58), and dysfunction in mitophagic pathways has been implicated in IBD pathogenesis (59, 60). In ulcerative colitis reduced mitochondrial function and higher cytokine secretion in the intestine is documented (61), and blocking cytokine signaling is an approved therapy for UC (62). However, molecular regulation of mitophagy dysfunction and its crosstalk with cytokine production in UC are not well established. In the colonic biopsy of patients with UC, we observed a smaller mitochondrial population, enhanced fission-inducing gene expression, and reduced mitophagy. Interestingly, only in the affected region of the colon, a drastic underexpression of CLUH protein (not RNA) was noticed in these patients. We observed a subpopulation of the patients (*n* = 6) with a higher CLUH mRNA level (albeit a similarly reduced protein level like the other patients). However, our patient cohort had only active patients (with UC DAI score more than 10), and these 6 patients could not be stratified in any subpopulation. We believe this might be due to heterogeneity in the population. CLUH degradation pathway was still active in these 6 patients, rendering a low CLUH protein level like the other patient groups. We next verified that CLUH protein is expressed in human colonic macrophages. Next, we documented in human primary macrophages that CLUH deficiency led to higher mitoROS, mitochondrial citrate, and mtDNA release in the cytosol after CLUH knockdown, all of which are involved in enhancing proinflammatory cytokine production (4, 18, 63, 64).

We thereby believe a reduction in CLUH level specifically in the affected region of the colon predisposes the patients to mitochondrial dynamics alteration and produces more disease-associated cytokines. Of interest, patients with UC have higher gut permeability, and bacterial ligands like LPS can reach to the mucosal cells easily (65). We provide evidence that LPS treatment after 2 hours can sumoylate and ubiquitinate CLUH and commit the cell to an inflammatory phenotype. It is also possible that CLUH expression can be transcriptionally regulated by other factors, and CLUH RNA level may also be altered at later time point of LPS stimulation. In the LPS-induced septic shock model, a reduction in murine CLUH RNA level has been demonstrated (66).

How does CLUH regulate cytokine production in the macrophages? Under CLUH knockdown, the enhanced cytokine production was abrogated to some extent after blocking mitoROS production, blocking glycolysis, blocking mitochondrial citrate release, or blocking mitochondrial DNA synthesis, indicating synergistic effect of all these pathways in CLUH-mediated cytokine production. Using Mdivi1 to inhibit the fission or drp1 pathway also had similar results. However, just enhancing mitophagy by 2,4 DNP had no effect on the cytokine production as the dysfunction prevailed as shown by the drp1 level and finally at the lysosomal degradation step. We thereby believe the effect of CLUH occurs at multiple nodes in human macrophages and in the intestinal mucosa. Our results are in line with the fact that mitoROS, mtDNA, and mitochondrial derived citrate released in the cytosol have been shown to enhance proinflammatory cytokine production (4, 18, 63, 64, 67). Further, mitochondrial morphology dictates the immune function as enhanced fission promotes TLR-induced IL-12 production in mouse bone marrow–derived macrophages and bone marrow–derived dendritic cells (68). Mitophagy inhibition also was previously shown to be involved in enhanced cytokine secretion during sepsis (20) and kidney fibrosis (19). We did not find any effect of CLUH in IL-1β production. This result was surprising as higher mtDNA release has been linked with NLRP3 inflammasome activation (69–71), but we assume that fissioned mitochondria might not favor NLRP3 inflammasome complex docking.

In conclusion, our study presents a concept of IBD immunology through modulation of mitochondrial dynamics. Increased mitochondrial density has proved to be a biomarker of sepsis in critically ill patients (20). Fission inhibitors and mitophagy activators have additionally been used to reduce disease pathogenesis of sepsis, kidney fibrosis, and specifically IBD in the animal model (9, 72, 73). We demonstrate function for an RBP in regulating mucosal innate immune function, which has profound implications for treatment of UC and other immune disorders. Therapeutics endowed with CLUH activators might help to address multiple mitochondrial immune disorders.

## Methods

### Cell culture and reagents.

Human monocyte THP1 cells and HEK293T cells were cultured in 4.5 g/L glucose DMEM/RPMI supplemented with 10% FBS (Gibco), 2% penicillin/streptomycin, and 2 mM l-glutamine (Invitrogen). PMA was used to convert the THP1 cells to macrophages. Cells were obtained from American Type Culture Collection at CSIR - Central Drug Research Institute (Lucknow, India). Reagents used are listed in Supplemental Table 3.

### Human blood MDM preparation.

Monocytes were purified from human peripheral blood mononuclear cells by positive CD33 (48) selection (Miltenyi Biotec) and cultured with 10 ng/mL macrophage colony-stimulating factor (GenScript) for 7 days as before (4). Macrophage polarization was done using LPS and IFN, as described before (4).

### siRNA transfection.

Dharmacon or Epigenetec SMARTpool siRNA for *CLUH* and *PolG* were purchased and electroporated (100 nM) into primary cells using Amaxa Nucleofactor according to the manufacturer’s instructions. Cells were incubated at 37°C for 24 hours prior to initiating assays or treatment. siRNAs used are listed in Supplemental Table 2.

### Protein expression analysis.

Western blot was performed using anti-CLUH, anti-PINK1, anti-Parkin, anti-LC3, anti-mTOR, anti–Drp-1, anti-COXIV, anti-DLST, anti-SDHA, anti-HK1, anti-PDH, anti-TOM20, and anti-VDAC for mitochondrial proteins and anti-NDP52, anti-Beclin1, and anti-BNIP3 for mitophagy markers using standard technique. Loading controls included anti-GAPDH and anti–β-actin. Proteins were immunoprecipitated using CLUH antibody using bound protein A-Sepharose beads. Antibodies used are listed in Supplemental Table 1.

### mRNA expression analysis.

RNA was isolated from cells or homogenized mice and human organ tissue, then reverse-transcribed, and quantitative PCR was performed (7500 Software v2.3), normalized to GAPDH/β-2-microglobulin. Primers used are listed in Supplemental Table 2.

### Microscopy.

MDMs were seeded at a density of 20,000 cells/well. Then mitochondria were stained using anti-MTCO2 (Abcam) or anti-TOM20 (Cell Signaling Technology). Lysosomes were stained with anti-LAMP1 (Cell Signaling Technology), and nuclei were stained with DAPI (Acros Organics). Fluorescence microscopy was conducted with FV10-ASW4.1 viewer and Leica SP5 DM microscope and analyzed with LASX software (Leica).

### H&E scoring and DAI scoring.

To study the histopathological changes, tissues were fixed in 10% formal saline solution. Paraffin sections (4 μm thick) were cut from formalin-fixed, paraffin-embedded tissue blocks, deparaffinized, rehydrated, and stained with H&E. The slides were visualized under light microscope (Leica DMi-5000 microscope). H&E score was assigned for inflammation, crypt alteration, and goblet cell loss, as before (74). DAI was noted for stool consistency, rectal bleeding, and body weight loss. The scores were given as follows: minimal change: <10%, 0–1 = normal, mild: 10–25%, 2 = mild, moderate: 26–50%, 3 = moderate, highest: >50%, 4 = severe. DAI scores were plotted for each mouse every day, as before (75).

### Immunohistochemical staining.

Sections (4 μm thick) were cut from formalin-fixed, paraffin-embedded tissue blocks, deparaffinized, and rehydrated in ethanol, and incubated with polyclonal antibody against CLUH (dilution 1:100) at 4°C overnight. Applied Primary Antibody Amplifier Quanto (Thermo Fisher Scientific) incubated for 10 minutes followed by incubation with HRP Polymer Quanto (Thermo Fisher Scientific) for 10 minutes. Signal was detected by color development with DAB as chromogen. Tissue sections were counterstained with hematoxylin and analyzed under a bright-field microscope (Nikon Eclipse E200).

### Glycolytic and citrate release, cathepsin B, acid phosphatase assay.

Glycolytic (565 nm, colorimetric) and citrate release (530–585 nm, fluorometric), cathepsin B (400-505 nm, fluorometric), and acid phosphatase activity (360–450 nm, fluorometric) were determined using respective microplate assay kits. Duplicates of each sample were measured using 20 μg protein amount as before (76).

### mitoROS production.

To measure superoxide levels, cells were treated with 100 nM MitoSOX (Life Technologies, Thermo Fisher Scientific) at 37°C for 30 minutes. Samples were assessed immediately by flow cytometry.

### Mitophagy and autophagy assays.

MDMs were transfected with mt-mKeima (Addgene, mKeima-Red-EB3-7, no. 56015) to analyze mitophagy at 406 nm and 561 nm by flow cytometry as before (19). Autophagy was measured using Cyto-ID assay kit as before (20).

### Mitochondrial assay.

Human macrophages were incubated with MitoTracker Deep Red and MitoTracker Green (Invitrogen) for 30 minutes. Cells were collected in PBS and acquired immediately by flow cytometry (BD Biosciences) as before (77).

### Analysis of cytokine levels.

Human MDMs were cultivated and activated as indicated during 24 hours. TNF-α, IL-10, IL-1β, and IL-6 cytokine levels were assayed in the cell supernatants as per the manufacturer’s (eBioscience) instructions.

### Quantification of mtDNA release.

For mtDNA quantification, total DNA was isolated from WT and CLUH-knockdown MDMs by using a DNeasy Blood and Tissues Kit (QIAGEN). Quantitative reverse-transcription PCRs were performed in triplicate in 96-well reaction plates (Applied Biosystems). Each reaction (final volume 10 μL) contained 25 ng DNA, 5 μL of Power SYBR-Green PCR Master Mix (Applied Biosystems), and 0.5 μM of each forward and reverse primer. Mitochondrial and nuclear encoded gene was amplified. Primers used are listed in Supplemental Table 2.

### Guide RNA oligo design and cloning.

Designed guide RNA oligos were synthesized from Sigma and annealed by incubation at 95°C followed by cooling. pL.CRISPR.EFS.PAC vector (pL-CRISPR.EFS.PAC from Addgene, no. 57828) was digested with Esp3I and gel purified. Digested vector backbone and annealed oligos were ligated using T4 DNA ligase and transformed. Colonies were screened for positive clones and validated for positive cloning by PCR and sequencing.

### Lentivirus production and titration.

HEK293T cells were seeded in a 10 cm dish and grown in DMEM and 10% FBS medium for 24 hours or until they achieved 70% confluence. Cells were transfected with lentivirus vector (Addgene, no. 13425) encoding shRNA together with helper plasmids (Addgene; pMD2.G, no. 12259; psPAX2, no. 12260) by using polyethylenimine (PEI). At 24 hours later, medium was changed and cells were further incubated for 48 hours. After 48 hours and later every 24 hours, medium was collected and viral particles were concentrated.

### Transduction.

THP1 cells were transduced with viral particles for 24 hours along with 10 μg/mL protamine sulfate. After 72 hours, cells were selected with 2 μg/mL puromycin for 48 hours or until complete cell death in no virus–transduced control cells. Puromycin-resistant cells were selected and analyzed for CLUH protein expression.

### Construction of CLUH clone and mutants and transfection.

Full-length CLUH plasmid was generated in pEGFP-N1 vector. K1257, K12, and S279-281A mutants were generated through site-directed mutagenesis (QuikChange II Site-Directed Mutagenesis Kit, Agilent), and the mutations were confirmed by sequencing. Cells were transiently transfected by Lipofectamine 3000 (Invitrogen). After 24 hours transfection cells were lysed to confirm the expression by Western blot. Sequences used are listed in Supplemental Table 2.

### DSS colitis mouse model development.

Colitis was induced by daily administration of 2% DSS (MW 30,000–40,000) in C57BL/6 male (7 week) mice for 7 days in drinking water. Animals were obtained from the National Laboratory Animal Centre at the CSIR - Central Drug Research Institute (Lucknow, India). The clinical parameters used to score the disease were weight loss, loose stools, and presence of bleeding. At the end of treatment, colons were processed for histological analysis and cytokine production of immune cells by ELISA.

### shRNA transduction in vivo.

Lentivirus expressing Cluh shRNA or control shRNA was administered i.p. to DSS or control mice using the virus dose of 5 × 10^6^ particles/mouse on day 7 before DSS administration to reduce the expression of the gene of interest.

### Intestinal macrophage isolation.

Intestinal lamina propria cells were isolated from colon biopsy (48, 78, 79) after collagenase treatment and purified using CD33 beads (Miltenyi Biotec). Single-cell suspension was analyzed via flow cytometry to check macrophage purity.

### Colonocyte culture.

Intestinal lamina propria cells were isolated from colonic resection specimens from control and DSS mice. Briefly, intestinal resections were washed and then cut into 1 mm^2^ pieces, then incubated in a buffer consisting of RPMI, 75 mg/mL collagenase type VIII (Sigma), DNase (Sigma), and HEPES for 45 minutes in a C shaker. We collected the supernatant by centrifugation at 13,000*g* at 4°C.

### C. elegans studies.

We employed WT strain of *C*. *elegans* N2 (Bristol) for conducting the experiments. The strain was cultured using standard protocols. Gene silencing was carried out employing the feeding protocol as described previously (80). To check the effect of *clu-1* knockdown upon mitochondrial content, we employed MitoTracker dye staining protocol as before (81). RNA isolation of the control worms (empty vector) as well as *clu-1* gene–silenced worms was followed by cDNA preparation and mRNA quantification using quantitative PCR. Briefly, young adult worms were harvested using M9 buffer and then washed 3 times using 0.2% DEPC-treated water.

### Human tissue samples.

Colonoscopy was done by trained gastroenterologists with a fiber optic video colonoscope using standard technique. An attempt was made in every patient to reach cecum and terminal ileum. We obtained 4–6 mucosal biopsies using standard biopsy forceps from obviously affected area and apparently normal looking area, each. The samples were collected and immediately preserved for future use. For controls, we collected colon biopsies from patients with irritable bowel syndrome (noninflamed colon) using the same technique.

### Biopsy section confocal microscopy.

Human colon tissue (fixed in tissue-freezing medium) was cut in 4 μm thick sections, and mitochondria were stained using anti-MTCO2 (Abcam); nuclei were stained with DAPI (Acros Organics). Confocal fluorescence microscopy was conducted with Leica SP5 DM microscope with LASX software (Leica).

### Biopsy section TEM.

Thin sectioning TEM technique was employed to study the mitochondrial morphology of the human colon specimens as described earlier (1). Tissue specimens were fixed in 2.5% glutaraldehyde and 4% paraformaldehyde in phosphate buffer (pH 7.4) followed by postfixation in 2% osmium tetroxide. After dehydration in a gradient series of ethanol and resin infiltration, specimens were embedded and polymerized at 60°C in Spurr Resin. Ultrathin sections (50–70 nm) were obtained using a Leica EM UC7 ultramicrotome, collected on copper grids, and double stained with lead citrate and uranyl acetate. Grids were observed at 100 kV under a JEOL JEM1400 TEM equipped with a Gatan bottom-mounted Orius CCD camera (43).

### Statistics.

Significance was assessed using 2-tailed Student’s *t* test or 1-way/2-way ANOVA. *P* < 0.05 was considered significant. Error bars are shown as SEM.

### Study approval.

All the work with animal and human samples was done after acquiring proper approval from the institutional animal ethics committee (IAEC) for mice (CSIR-CDRI, Lucknow, India) and institutional ethics committee (IEC) for humans (CSIR-CDRI and SGPGI, Lucknow, India).

## Author contributions

S Khan, DR, SS, A Naseer, NCS, S Kumari, SI, JS, and PL designed, performed, and analyzed data from the experiments. MNM, AKT, MS, TC, VB, MKB, SKG, KM, A Nazir, UCG, and AL designed experiments and analyzed data. S Khan and AL wrote the manuscript. AL supervised the study and obtained funding.

## Supplementary Material

Supplemental data

## Version 1 05/04/2023

In-Press Preview

## Version 2 06/08/2023

Electronic publication
