# Supplementary material for: CLUH functions as a negative regulator of inflammation in human macrophages and determines ulcerative colitis pathogenesis
Source: JCI Insight. 2023 Jun 8;8(11):e161096. doi: 10.1172/jci.insight.161096 (PMC10393232; doi:10.1172/jci.insight.161096)
Supplement: Supplemental data [file jciinsight-8-161096-s199.pdf]

Supplementary Figures:

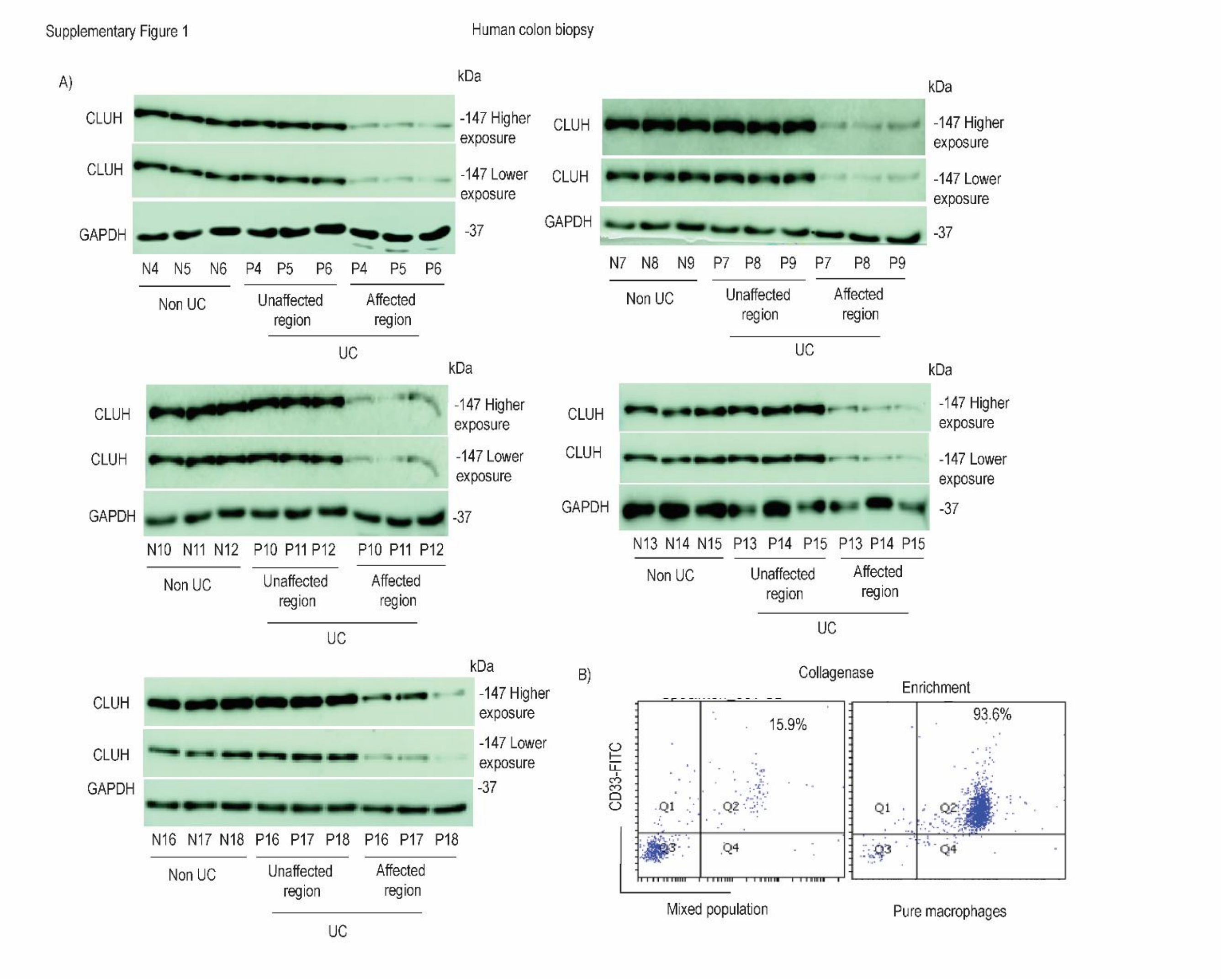

Fig 1: CLUH is expressed in human colon biopsy samples. (**A**) Western blot for CLUH expression with GAPDH as loading control is shown for 15 colon biopsy from non-UC control (N denotes non-UC, number denotes donor identification number), UC unaffected region (P denotes patient-UC, number denotes donor identification number) and UC affected region (P denotes patient-UC, number denotes donor identification number). (**B**) Representative flow cytometry for collagenase treated fresh biopsy samples; mixed and purified macrophages.

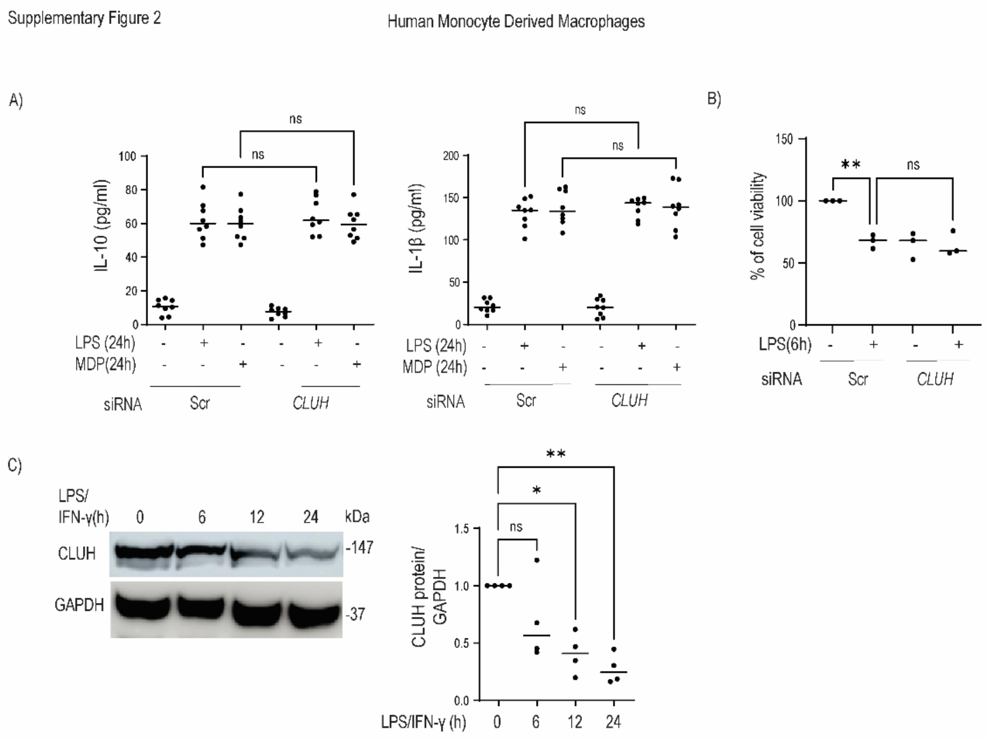

Fig 2: CLUH knockdown had no effect on IL1-β and IL-10 secretion. (**A**) MDMs were transfected with scrambled or *CLUH* siRNA for 24 h and then treated for 24 h with 100 ng /ml LPS or 100 ug /ml MDP and IL-10 and IL-1β secretion was measured from the cell supernatant. (n=8; with similar result observed in an additional n=8 donors). (**B**) MDMs were transfected with scrambled or *CLUH* siRNA for 24 h and then treated for 6 h with 250ng /ml LPS and incubated with MTT. Shown is MTT absorbance (570 nm) as a measure of cell viability. Hydrogen Peroxide (0.25Mm for 1 h) treated cells is used as a positive control of cell death induction. (**C**) MDMs were treated with IFN-γ 20ng /ml along with 100 ng /ml LPS for the indicated time and assessed for CLUH protein expression by western blot with GAPDH as loading control with a summary graph of a densitometry in which samples are normalized to GAPDH (n=4 donors). Mean + s.e.m; 'ns' denote non-significant; *P<0.05; **P<0.01; as determined by One-way ANOVA analysis.

Supplementary Figure 3

Human Monocyte Derived Macrophages

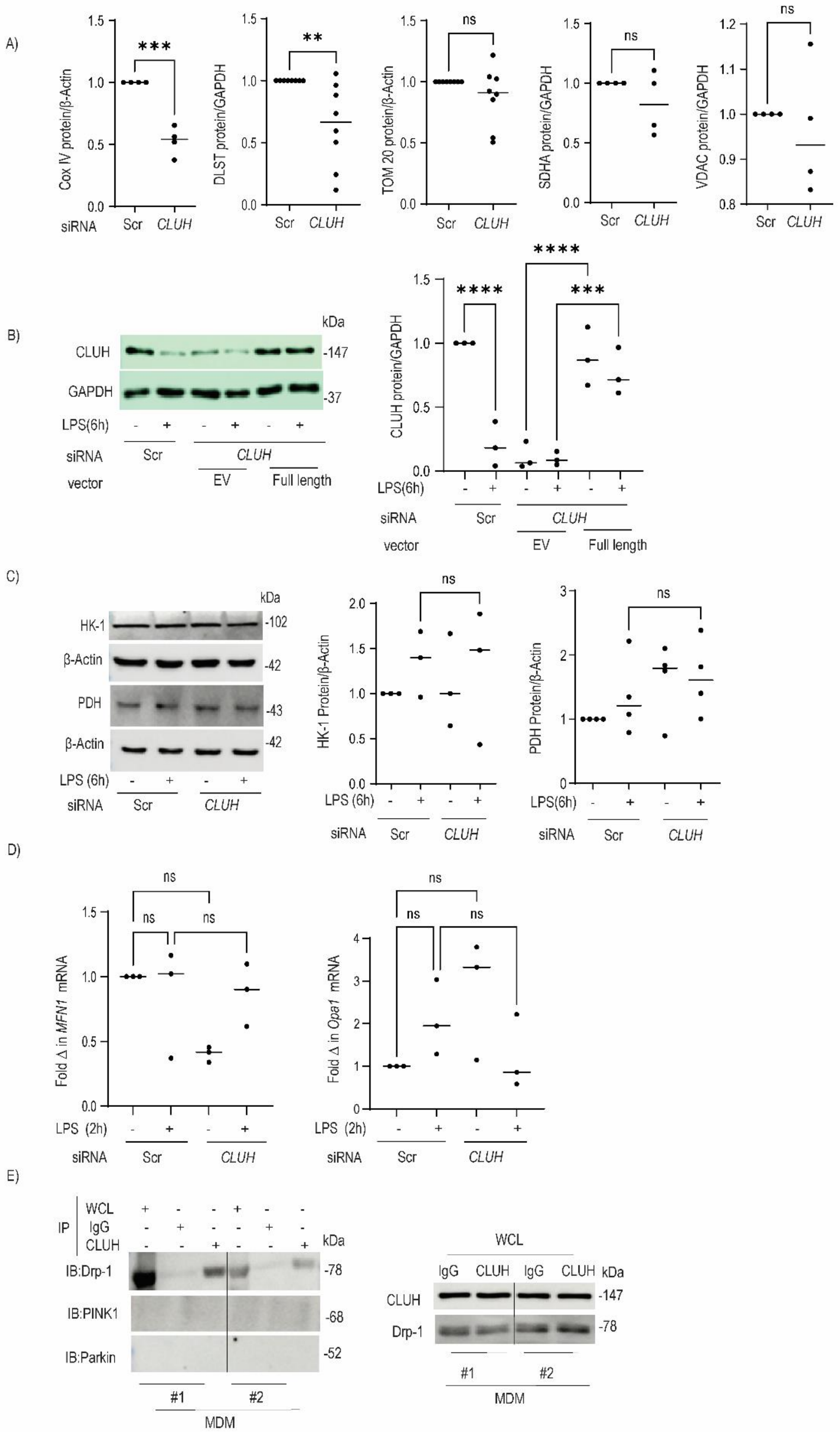

Fig 3: LPS treatment leads to CLUH mediated mitochondrial dysfunction and early glycolytic reprogramming. (**A**). Summary graph of densitometry for performed western blots for different mitochondrial protein expression such as COX IV, DLST, TOM20, VDAC, SDHA using GAPDH and β-Actin as loading control are shown (n=4 donors; for DLST and TOM20 N=8 donors). Full length CLUH plasmid (pEGFP-N1 vector) was also ectopically expressed in the CLUH knockout cells to rescue CLUH knockout condition. These cells were next treated with 100 ng /ml LPS for 6h and assessed (**B**) For CLUH protein expression by western blot with GAPDH as a loading control along with a summary graph of a densitometry in which samples are normalized to GAPDH (n=3). Human MDMs were transfected with scrambled or *CLUH* siRNA for 24 h and treated with 100 ng /ml LPS for 6 h and assessed for (**C**) Glycolysis pathway for HK1 and PDH protein expression were assessed by western blot with β-Actin as a loading control along with a summary graph of a densitometry in which samples are normalized to β-Actin (n=3 and n=4 donors respectively). (**D**) mRNA expression of mitochondrial fusion marker MFN1 and Opa1 after normalization with GAPDH after different time point of LPS treatment (n=3; with similar result for an additional n=6 donors). (**E**) CLUH was immunoprecipitated (IP) from MDM cell lysates and the recruitment of Drp-1, PINK and Parkin proteins were assessed by western blot (IB) (Data from n=2 donors is shown, with an additional n=4 with similar result). IgG pool down was used as IP control. Equal loading of the samples was confirmed by immunoblotting CLUH and Drp-1 proteins from all the samples run in a different gel. WCL, whole cell lysate. Mean+ s.e.m; 'ns' denote non-significant; and 'EV' denotes Empty Vector; 'ns' denote non-significant;**P<0.01; ***P<0.001; **** P<0.0001 as determined by 2 tailed t-test (For Supplementary Fig.3A) and One-way ANOVA analysis for the rest of the figures.

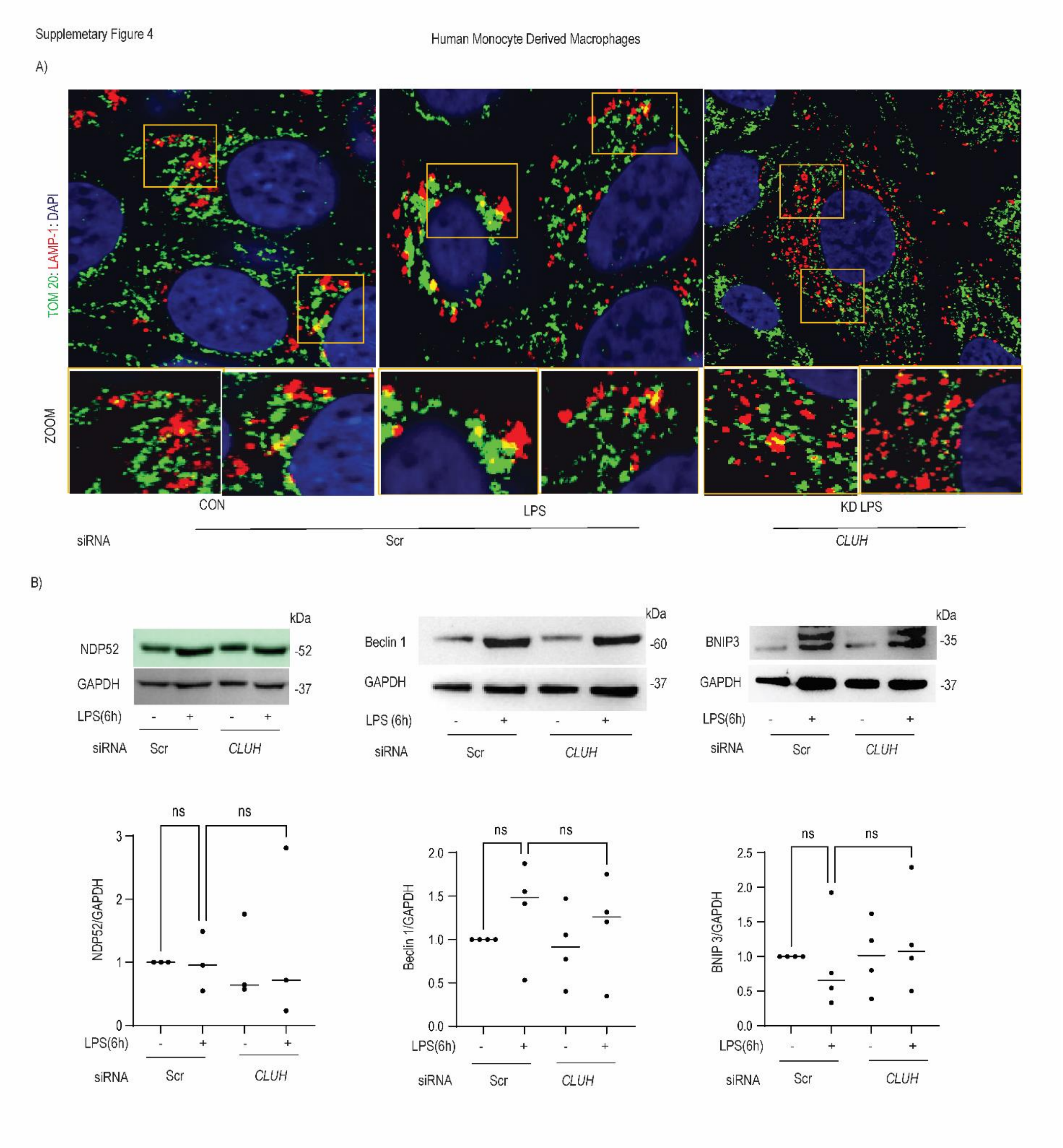

Fig 4: After CLUH knockdown, mitochondria-lysosome relative positioning and mitophagy marker expressions were not altered.

MDMs were transfected with scrambled or *CLUH* siRNA for 24 h and treated with 100 ng /ml LPS for 6 h and assessed (**A**) staining of Lysosome with LAMP-1 (red), mitochondria with TOM 20 (green), and nuclei with DAPI (blue). Yellow colour indicates colocalization of lysosome with mitochondria. (**B**) Western blot for NDP52, Beclin-1, BNIP3 with GAPDH as

a loading control is shown along with a summary graph of a densitometry in which samples are normalized to GAPDH (n=3). Mean+ s.e.m; ‘ns’ denote non-significant; and ‘EV’ denotes Empty Vector.

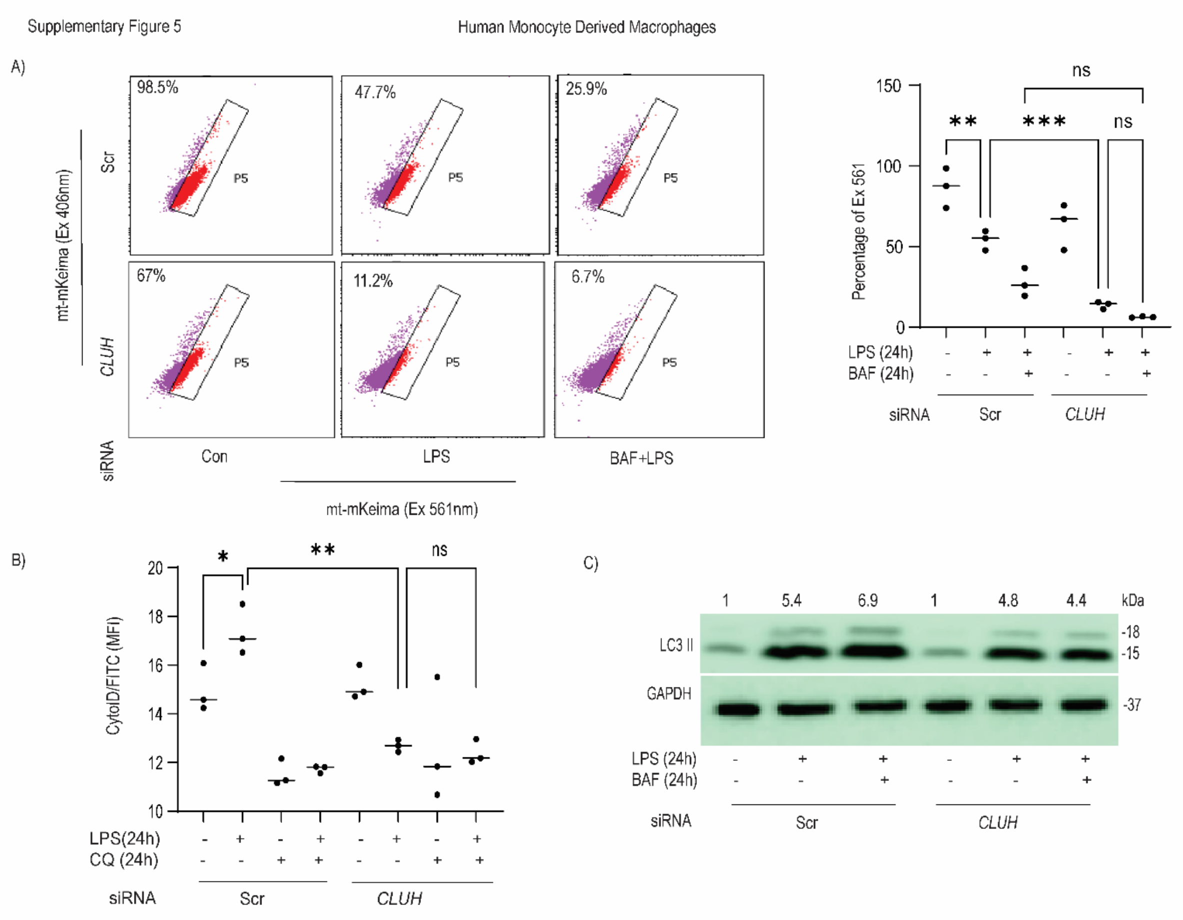

Fig 5: Lysosome inhibition had no added effect on mitophagy and autophagy after CLUH knockdown. (**A**) MDMs were transfected with scrambled or *CLUH* siRNA for 24 h and transfected with mt-mKeima plasmid and assessed for mitophagy by Flow cytometry at Ex406/Ex561. Representative and summarized Flow cytometry with the mean fluorescent intensity (MFI) value shown (n=3 samples). MDMs were transfected with scrambled and *CLUH* siRNA for 24 h and treated with 100 ng /ml LPS for 24 h along with the stimulation of 50 uM chloroquine 24 h and assessed for (**B**) Autophagy using flow cytometry after CytoID (green) staining. Representative and summarized flow cytometry with the mean fluorescent intensity (MFI) value shown (n=3; similar result for an additional n=3 donors). MDMs were transfected with scrambled or *CLUH* siRNA for 24 h and treated with 100 ng /ml LPS for 24 h along with 1uM Bafilomycin A1 and (**C**) LC3 II expression was checked with GAPDH as a

loading control along with a summary graph of a densitometry in which samples are normalized to GAPDH (with similar results in an additional 2 donor). Densitometry values are indicated above each band. Mean + s.e.m; ‘ns’ denote non-significant; ‘EV’ denotes Empty Vector; *P<0.05; **P<0.01; ***P<0.001 as determined by One-way ANOVA analysis.

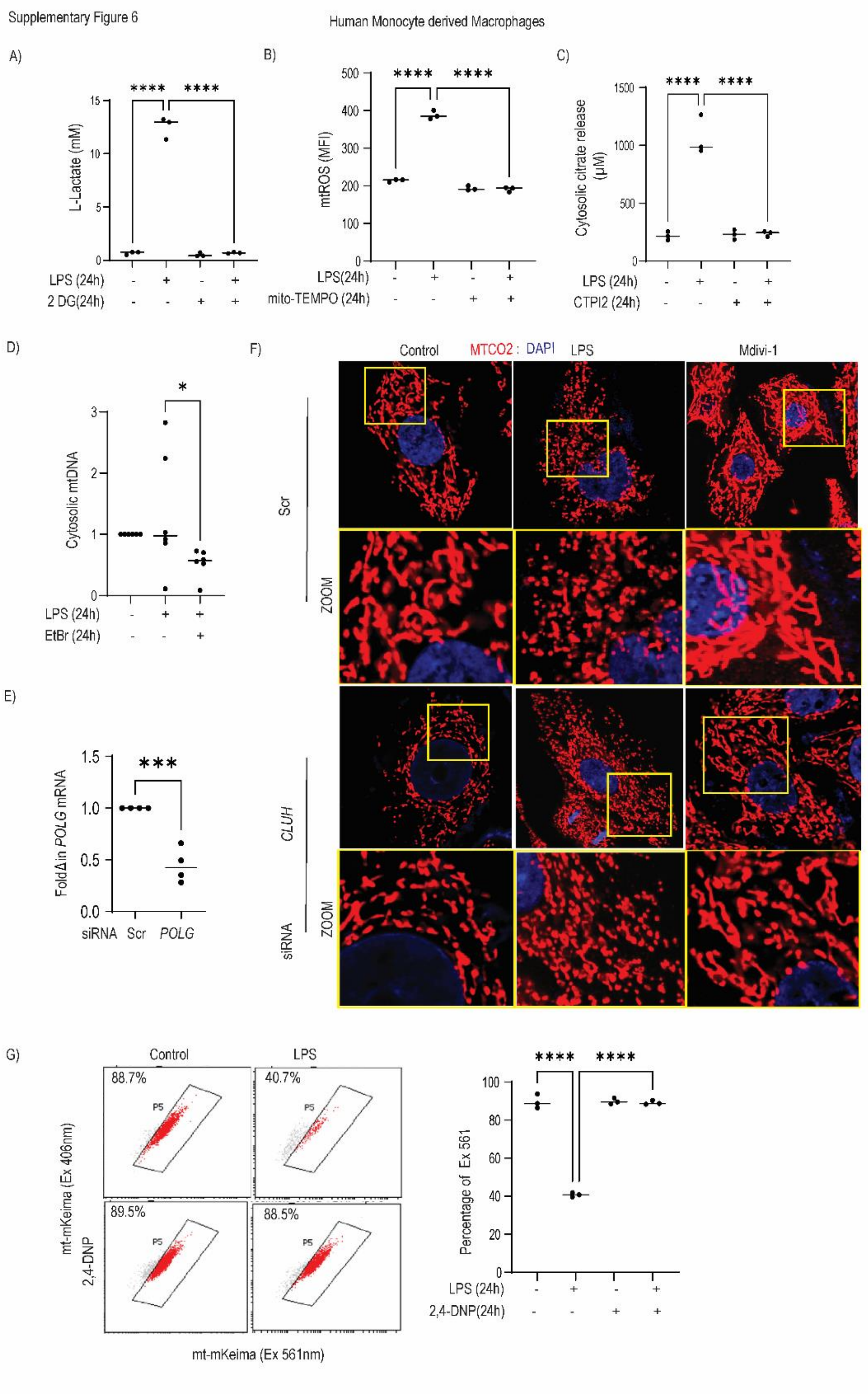

Fig 6: Validation of the siRNA and inhibitors used in this study: MDMs were treated with 100 ng /ml LPS and assessed for (**A**) Lactate release with or without 2-Deoxy-D-Glucose (2DG,5 mM, to inhibit glycolysis) (**B**) Assessed for mitoROS production with or without Mito-TEMPO (1 µM, to inhibit mtROS production) (**C**) assessed for citrate export to cytosol

with or without CTPI2 (1mM, to inhibit mitochondrial citrate transporter) (**D**) Checked for mitoDNA release in the cytosol with or without EtBr for 24 h (50 ng /ml to reduce mtDNA quantity) (**E**) Human MDMs were transfected with scrambled or mitochondrial DNA polymerase *POLG* siRNA for 24 h and assessed for mRNA expression of *POLG* after normalization with GAPDH (n=4 donors). (**F**) MDMs were treated with 100 ng /ml LPS along with Mdivi-1 (50 µM, to inhibit mitochondrial fission) for 24h and mitochondrial fission was checked by confocal microscopy. (**G**) MDMs were transfected with mt-mKeima plasmid and treated with 100 ng /ml LPS along with 2,4-DNP (50 µM, to activate mitophagy) for 24 h and checked for mitophagy activation at Ex406/Ex561by flow cytometry. Mean + s.e.m; *P<0.05; ***P<0.001; **** P<0.0001 as determined by 2 tailed t-test (Supplementary Fig. 6. E) and One-way ANOVA analysis for the rest of the figures.

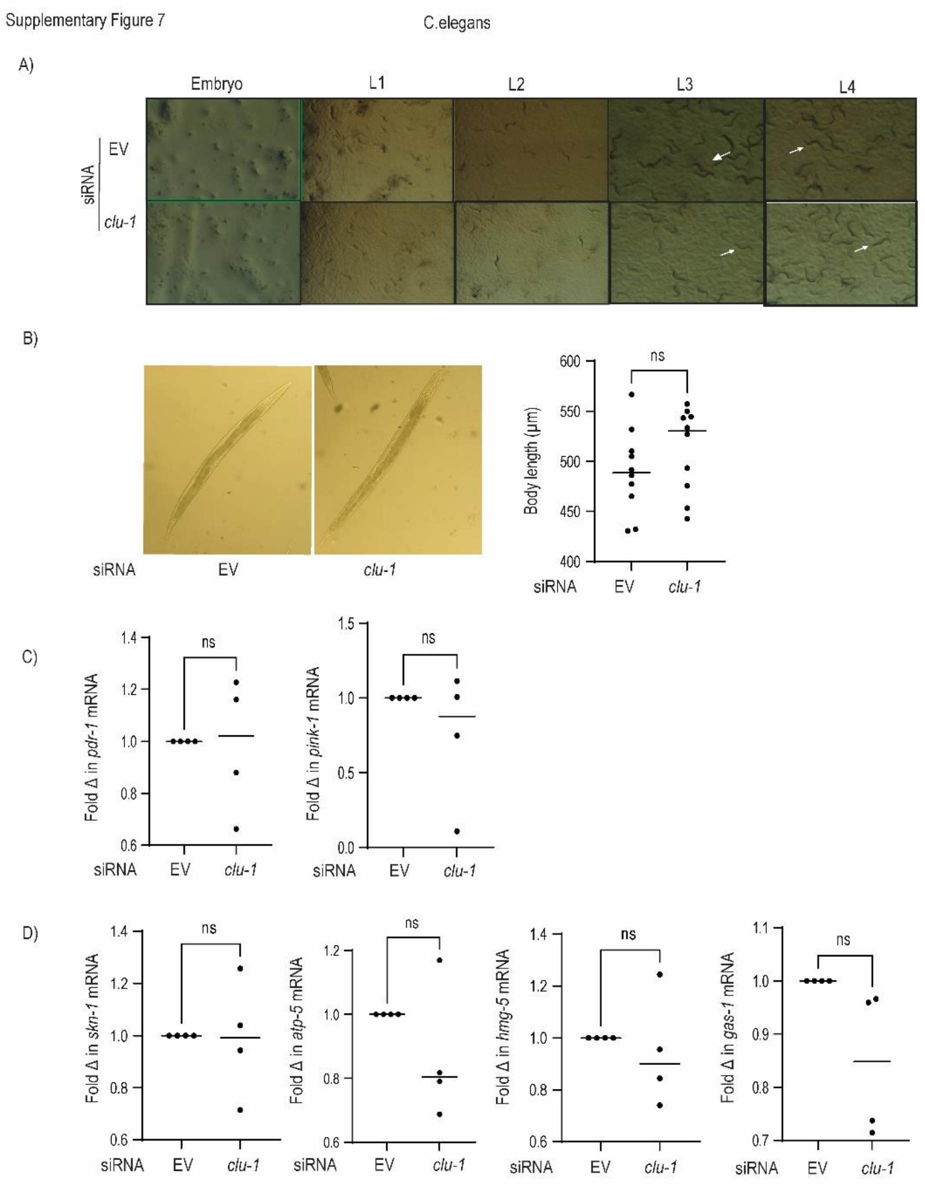

Fig 7: *Clu1* knockdown worms have no developmental defect and displayed no change in mitophagy and mitochondrial biogenesis markers. N2 strain of *C. elegans* were fed with HT115 bacteria carrying an empty vector or siRNA *clu*-1 for 24h and assessed for (**A**) Developmental stages (embryos->L1->L2->L3->L4) (**B**) Body morphology with the graphical representation (10 fields for each conditions) of relative change in the body length of young adult or L4 staged worms. (**C-D**) mRNA expression of mitochondrial mitophagy markers pdr-1 and pink-1 and mitochondrial biogenesis markers skn-1, atp-5,

hmg-5, gas-1 are shown after normalization with *β-Actin*. ‘EV’ denotes Empty Vector; ‘ns’ denote non-significant; when compared to the control worms.

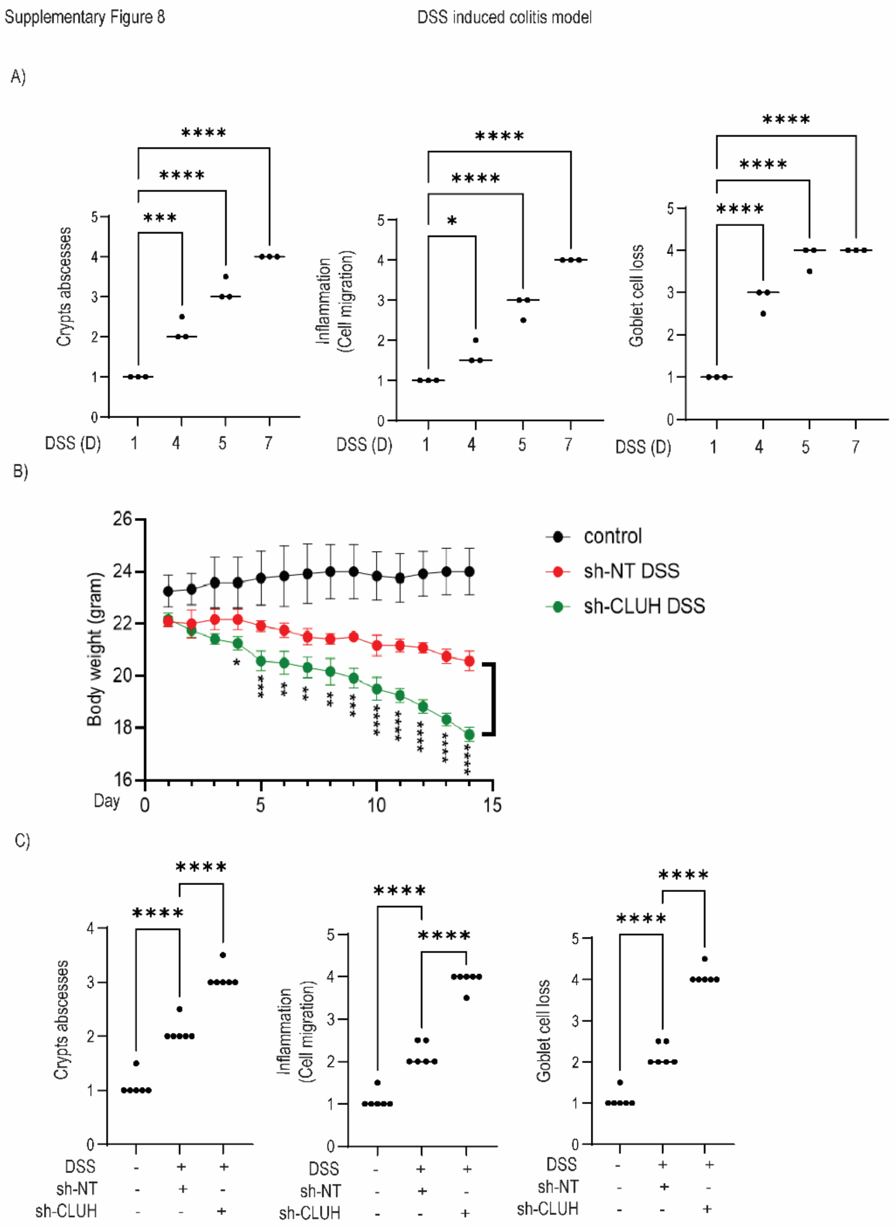

Fig 8: *Cluh* knockdown in mice exacerbates disease pathology in DSS induced colitis model. C57BL/6 male mice (7 weeks old) were fed Dextran Sodium Sulphate (DSS, 2.5%) in drinking water (n=5) for 7 days and assessed for (**A**) Hematoxylin and Eosin scoring in a day dependent manner (1day, 4days, 5 days, 7 days) including crypts abscesses, inflammation (cell migration), goblet cells loss. *Cluh* shRNA or non-targeted shRNA expressing lentivirus was administered via intraperitoneal route (5 X 10^6 particles /mouse on 7 and 1 day before DSS administration) to reduce the expression of CLUH and

checked for (**B**) Body weight in the three groups. (**C**) Hematoxylin and Eosin scoring including crypts abscesses, inflammation (cell migration), goblet cells loss in the three groups. Mean + s.e.m; ‘ns’ denote non-significant; **P<0.01; ***P<0.001; **** P<0.0001 as determined by One-way and Two-way ANOVA analysis.

Supplementary Figure 9

A)

No.	Pos.	Group	Score
1	K1257	KDLEN LKAE VARRH	0.91
2	K12	DCPES LKKE AAAAE	0.91
3	K995	VQQGF LKEG CELIN	0.73
4	K936	LREIS LKTG IQVLL	0.73
5	K1301	SQPPA AKDP SPSVQ	0.69
6	K810	QLDHV FKIG IGELI	0.68
7	K1170	SALQH EKEG YTIYK	0.33
8	K174	GDSGK RKKG LEMDP	0.27
9	K295	ISPTF KKNF AVLQK	0.13

B)

Human	PASPRFLsHsLVELL	S271-S281
Mouse	PASPRFLSHSLVEL	S271-S281
RAT	PASPRFLSHSLVELL	S271-S281

Fig 9: Sumoylation/Ubiquitination sites in CLUH. (**A**) SUMOylation site prediction in CLUH through SUMOplot analysis program and the probability scores are shown in red. (**B**) Multiple protein sequence alignment of CLUH fragment from Human, Mouse and Rat. The conserved serine residues are shaded (red) using the consensus criteria.

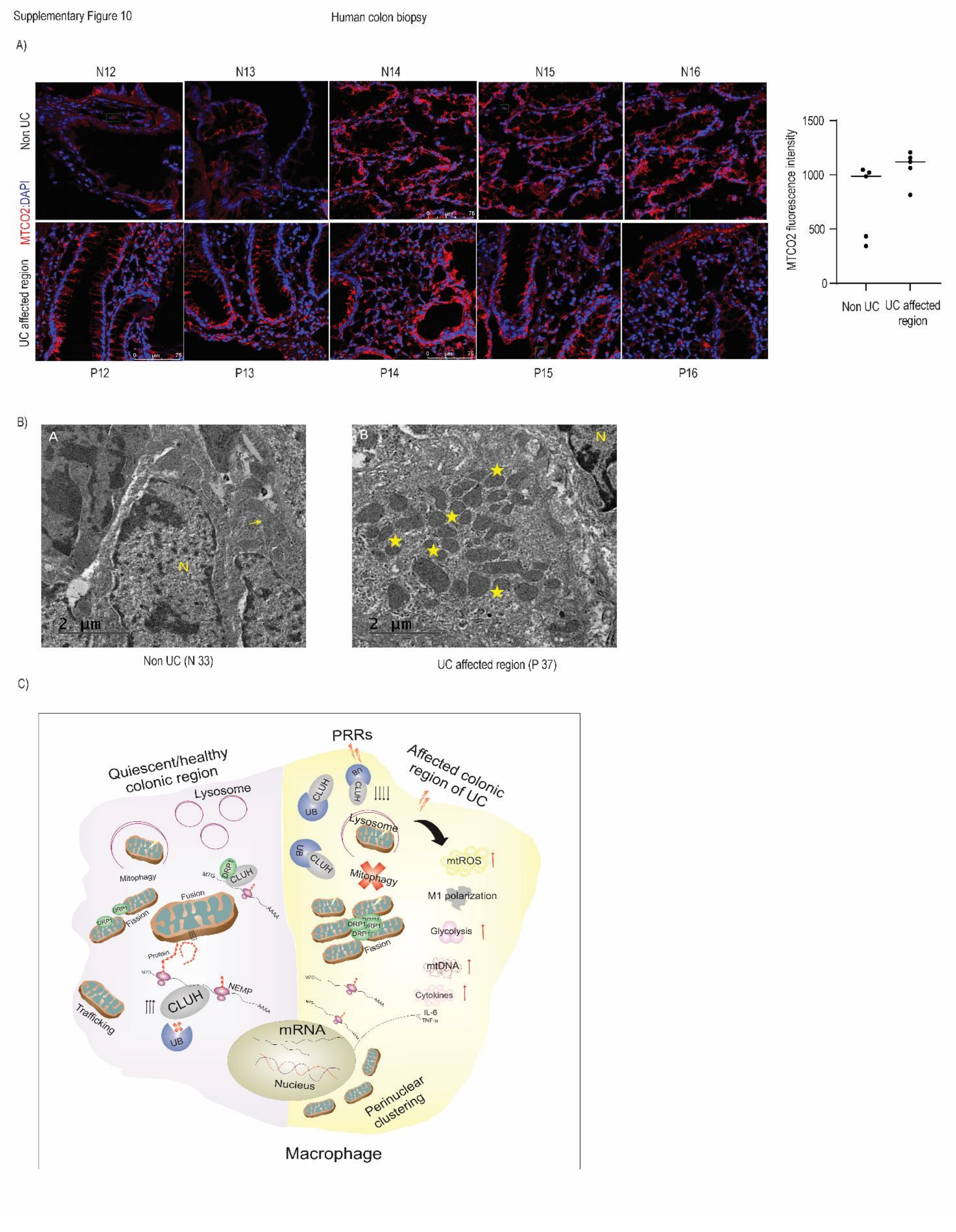

Fig 10: Mitochondrial density is high in the UC patients. (A) Human colonic biopsy tissues of non UC control (N denotes non-UC, number denotes donor identification number) and affected region of UC patients (P denotes patient-UC, number denotes donor

identification number) were stained with mitochondrial marker MTCO2 (Red) and DAPI to stain nucleus (blue). Summary graph of MTCO2 fluorescence intensity is shown. (**B**) Representative Transmission Electron micrographs of thin sections of human intestinal tissue of healthy (non UC) and affected region of ulcerative colitis (UC) patient. Numerous fragmented mitochondria (asterisks) were observed in the UC patient tissue elongated mitochondria in healthy tissue. N=nucleus. (**C**) Pictorial model depicting role of CLUH in mediating PRR-induced outcomes. Upon PRR stimulation, CLUH undergo degradation, thereby decreasing CLUH level, resulting in reduced protein targeting to the mitochondria which leads to mitochondrial dysfunction. This is associated with increased Drp-1 transcription. CLUH also binds to Drp-1 directly and sequesters it away from mitochondria. In the absence of CLUH, these function ultimately enhances mitochondrial fission, however with a reduced mitophagy, due to lysosomal dysfunction. Decreased CLUH expression during PRR stimulation leads to increased mtDNA, citrate release, glycolysis enhancement as well higher pro-inflammatory cytokine production from human macrophages and in the UC patients.

NAME	COMPANY	CAT. NO.	DILUTION

Supplementary Tables:

1. **Antibody**

**CLUH**	ABclonal	A10140	1:1000-1:4000
**NDP52**	CST	60732	1:1000
**Beclin 1**	CST	3495	1:1000
**BNIP-3**	CST	44060	1:1000
**CLUH**	abcam	ab222075	1:1000
**CD-33**	CST	77576	1:500-1:2000
**PINK**	CST	6946	1:1000
**PARKIN**	CST	4211	1:1000
**LC-3**	CST/sigma	3868/L8918	1:1000
**TOM-20**	CST	42406	1:1000
**CYTO-C**	CST	4280	1:1000
**COX-IV**	CST	4850	1:1000
**SDHA**	CST	11998	1:1000
**DLST**	CST	11954	1:1000
**HK-1**	CST	2024	1:1000
**PDH**	CST	3205	1:1000
**ACTIN**	CST	4967S	1:1000
**GAPDH**	CST	5174	1:1000
**MTCo2**	Abcam	Ab3298	1:300
**LAMP-1**	Novus	NB120-19294	1:1000
**VDAC**	CST	4661	1:1000
**mTOR**	CST	2983	1:1000
**pMTOR**	CST	5536	1:1000
**DRP-1**	CST	5391	1:1000
**Alexa Flour Goat anti Rabbit**	THERMO FISHER SCIENTIFIC INVITROGEN	A-11008	1:300
**Alexa Flour goat anti Mouse**	THERMO FISHER SCIENTIFIC INVITROGEN	A28175	1:300
**Quanto**	THERMO FISHER SCIENTIFIC	TL015QHD	1:100
**HRP Polymer Quanto**	THERMO FISHER SCIENTIFIC	TL015QHD	1:300

2. Primers

Name	Company	Sequence
**CLUH(Hu)**	SIGMA	F :- CCCGCCACCATGGTTATCAA<br>R :- GACTGATGGCAGCTCGGC
**DRP-1**	SIGMA	F :- GCCTCAGATCGTCGTAGTGG<br>R :- TCCATGTGGCAGGGTCATTT
**MFN-1**	SIGMA	F:- CAGGGACGGAGTGAGTGTC<br>R :-TTCTGCCATTATGCACCTGG
**OPA-1**	SIGMA	F:- CTGCAGGTCCCAAATTGGTT<br>R:- TCTTTGTCTGACACCTTCCTGT
**B2M**	SIGMA	F :- TGACTTTGTCACAGCCCAAG<br>R :- AGCAAGCAAGCAGAATTTGG
**skn-1**	SIGMA	F :- TACAGAACGTCCAACCACATC<br>R :- GCCCTTCTCTCCAGCAATATC
**atp-5**	SIGMA	F :- TCGAGTATTTCCCAGCTCATTTC<br>R :- CACGTCGAAGAACCTTGTAGTC
**PINK**	SIGMA	F :- TTGCAATGCCGCTGTGTATG<br>R :- TGGAGGAACCTGCCGAGATA
**PARKIN**	SIGMA	F :- GAGTCCAGGAGCTTGACACG<br>R :- TGTAGGTGGGTTTAACTGGACC
fzo-1	**SIGMA**	F :- TTCCGAAGAACAGGCAATGA<br>R :- TCTCCAACCAACAGCCTTATAC
**gas-1**	SIGMA	F :- CGTGAGAAAGACCGAACCATAC<br>R :- CTCCTCAATACGGCACAAGTATC
**EAT-3**	SIGMA	F :- GTCATACAACACCTCGGACAA<br>R :- CCAGATCCTCTGGGAAAGATTC
**hmg-5**	SIGMA	F :- AGCGGAAAGAGCAAGTAGAC<br>R :- TTCCAGCTCCAGACAACTTC
**MFF-1**	SIGMA	F :- GGTGCCGACCTTATGCAGAT<br>R :- GACTTCCATTCTGAGTGACGTAGT
**GAPDH(Hu)**	SIGMA	F :-TCGGAGTCAACGGATTTGGT<br>R :-TTCCCGTTCTCAGCCTTGAC

**pink-1**	SIGMA	F :- AGTCGTCTGGACAAAGTGATG<br>R :- TTGCTCGAAGTTGTCGTTCT
**Actin(C.E)**	SIGMA	F :- GGAGAGTGTTTCCTCGTCCC<br>R :- ATGAAGGGGTCGTTGATGGC
**pdr-1**	SIGMA	F :- CAGACGTCGTACAGCGAATAC<br>R :- TCATAGGGCTCCCAGAAGAA
**Clu-1**	SIGMA	F :- GGCAAGAAACGAGTTACATCTG<br>R :- AAGGGAGTCGGCCTCAATCGGG
**POLB**	SIGMA	F:- CATGTCACCACTGGACTCTGCAC<br>R:- CCTGGAGTAGGAACAAAAATTGCTG
**mitochondrion**	SIGMA	F:- TTTCATCATGCGGAGATGTTGGATGG<br>R:- TCTAAGCCTCCTTATTCGAGCCGA

3. Chemicals

Name	Company	Cat no.	Working Concentration
mitoTEMPO	Santa cruz	Sc221945	1 um
Mdivi-1	SIGMA	M0199	50um
Lipofectamine 3000 reagent	INVITROGEN	L3000001	
M-CSF, Human	GENSCRIPT	Z02914	10ng/ml
LIPOPOLYSACCHARIDE(LPS)	SIGMA	L2654	250ng/ml
MS COLUMNS	MILTENYI BIOTEC	130-042-201	
3,3’,5,5’-Tetramethylbenzidine (TMB Substrate)	SIGMA	T5525	
Poly(I:C) HMW	INVIVOGEN	Tlrl-pic	10ug/ml
TRIzol Reagent	INVITROGEN	15596026	
RPMI-1640 Media	GIBCO THERMO FISHER SCIENTIFIC INVITROGEN	22400071	
DMEM Media High glucose	GIBCO THERMO FISHER SCIENTIFIC INVITROGEN	11965126	
Fetal Bovine Serum (FBS)	GIBCO THERMO FISHER SCIENTIFIC INVITROGEN	26140079	
Trypsin EDTA	GIBCO THERMO FISHER SCIENTIFIC INVITROGEN	25200072	
Phosphate Buffer Saline (PBS) 10X	SIGMA	P7059-1L	
Mitosox	INVITROGEN	M36008	5 um
MG-132	SIGMA	M8699	50um
MitoTracker Deep Red	INVITROGEN	M22426	200nm
MitoTracker Green	INVITROGEN	M7514	200nm
Ethidium Bromide	SIGMA	E1510	10ug/ml
2,4-DNP	SIGMA MERCK	D198501	50um
MTT	THERMO FISHER SCIENTIFIC	M6494	1mg/ml
DAPI	SIGMA	D9542-10Mg	
DMSO Molecular Grade	SIGMA	D8418	
DPX mount for histology	SIGMA	06522	
H2O2	SIGMA	349887	0.25mm

Dextran Sulfate Sodium (DSS)	SIGMA	42867-25G	2.5%
Haematoxylin	SIGMA	H9627-100G	
RIPA Buffer 10X	SIGMA	20-188	
Eosin	SIGMA	318906-500ML	
Protease Inhibitor Cocktail	SIGMA	P8340-1ML	
Xylene	SIGMA	534056-4L	
RBC Lysis Buffer	SIGMA	11814389001	
2-Propanol (Isopropanol)	SIGMA	19516-500ML	
Histopaque	SIGMA	10771-100ml	
DAB	SIGMA	D3939	
Nematode growth medium	US-BIOLOGICAL LIFE SCIENCE	N1000	
Takara master mix (TB Green® Primer Ex Taq™)	TAKARA	RR420A	
Tween-20	SIGMA	P9416-100ML	
SODIUM DODECYL SULFATE (SDS)	SIGMA	L3731-1KG	
TRIZMA BASE	SIGMA	T1503-1KG	
GLYCINE	SIGMA	G8898-1KG	
ACRYLAMIDE	SIGMA	A887-1KG	
TEMED	SIGMA	T9281-100ML	
AMMONIUM PER SULFATE (APS)	SIGMA	A3678-100GM	
SODIUM-AZIDE	SIGMA	71289	
2-Deoxy-D-glucose (2DG)	SIGMA	D8375-10GM	5mm
CD33 MICRO BEADS HUMAN	MILTENYI BIOTEC	130050201	
CHLOROFORM	SIGMA	C2432-1L	
ETHANOL	SIGMA MERCK	02870	
Proteinase	SIGMA	10165921001	
L-15medium	SIGMA	L1518	
Nile-red dye	MP BIOMEDICAL	151744	
H_2DCFDA (2',7'-dichlorodihydrofluorescein	SIGMA	35845-1G	

diacetate)			
MDP	INVIVOGEN	tlrl-mdp	10ug/ml
SYBER GREEN	APPLIEDBIOSYSTEMS	A25742	
HIGH-CAPACITY cDNA reverse transcription kit	APPLIEDBIOSYSTEMS	4368814	
Penicillin-Streptomycin	Sigma	P4333-100ml	
CTPI 2	Selleck chemicals	S2968	1mm
mt-mKeima	Addgene	56018	
CYTO ID	Enzo	ENZ-51031	
